# Association of the tomato co-chaperone gene *Sldnaj* harboring a promoter deletion with susceptibility to Tomato spotted wilt virus (TSWV)

**DOI:** 10.1093/hr/uhaf019

**Published:** 2025-01-15

**Authors:** Shiming Qi, Liang Zhe Meng, Qianqi Lou, Yushun Li, Yuanbo Shen, Shijie Zhang, Xinyu Wang, Pan Zhao, Jin Wang, Bo Wang, Xiubin Chen, Chunmei Zhang, Yu Du, Jiantao Zhao, Xiangqiang Zhan, Yan Liang

**Affiliations:** State Agriculture Ministry Laboratory of Northwest Horticultural Plant Germplasm Resources & Genetic Improvement, College of Horticulture, Northwest A&F University, Yangling, Shaanxi 712100, China; College of Agriculture and Ecological Engineering, Hexi University, Zhangye, Gansu 734000, China; State Agriculture Ministry Laboratory of Northwest Horticultural Plant Germplasm Resources & Genetic Improvement, College of Horticulture, Northwest A&F University, Yangling, Shaanxi 712100, China; State Agriculture Ministry Laboratory of Northwest Horticultural Plant Germplasm Resources & Genetic Improvement, College of Horticulture, Northwest A&F University, Yangling, Shaanxi 712100, China; State Agriculture Ministry Laboratory of Northwest Horticultural Plant Germplasm Resources & Genetic Improvement, College of Horticulture, Northwest A&F University, Yangling, Shaanxi 712100, China; State Agriculture Ministry Laboratory of Northwest Horticultural Plant Germplasm Resources & Genetic Improvement, College of Horticulture, Northwest A&F University, Yangling, Shaanxi 712100, China; State Agriculture Ministry Laboratory of Northwest Horticultural Plant Germplasm Resources & Genetic Improvement, College of Horticulture, Northwest A&F University, Yangling, Shaanxi 712100, China; State Agriculture Ministry Laboratory of Northwest Horticultural Plant Germplasm Resources & Genetic Improvement, College of Horticulture, Northwest A&F University, Yangling, Shaanxi 712100, China; State Agriculture Ministry Laboratory of Northwest Horticultural Plant Germplasm Resources & Genetic Improvement, College of Horticulture, Northwest A&F University, Yangling, Shaanxi 712100, China; State Agriculture Ministry Laboratory of Northwest Horticultural Plant Germplasm Resources & Genetic Improvement, College of Horticulture, Northwest A&F University, Yangling, Shaanxi 712100, China; State Agriculture Ministry Laboratory of Northwest Horticultural Plant Germplasm Resources & Genetic Improvement, College of Horticulture, Northwest A&F University, Yangling, Shaanxi 712100, China; College of Agriculture and Ecological Engineering, Hexi University, Zhangye, Gansu 734000, China; College of Agriculture and Ecological Engineering, Hexi University, Zhangye, Gansu 734000, China; State Agriculture Ministry Laboratory of Northwest Horticultural Plant Germplasm Resources & Genetic Improvement, College of Horticulture, Northwest A&F University, Yangling, Shaanxi 712100, China; Boyce Thompson Institute for Plant Research, Ithaca, NY 14853, USA; State Agriculture Ministry Laboratory of Northwest Horticultural Plant Germplasm Resources & Genetic Improvement, College of Horticulture, Northwest A&F University, Yangling, Shaanxi 712100, China; State Agriculture Ministry Laboratory of Northwest Horticultural Plant Germplasm Resources & Genetic Improvement, College of Horticulture, Northwest A&F University, Yangling, Shaanxi 712100, China

## Abstract

Tomato spotted wilt virus (TSWV) poses a significant threat as a devastating pathogen to the global production and quality of tomato (*Solanum lycopersicum*). Mining novel resistance genes within the tomato germplasm is an effective and environmentally friendly approach to combat TSWV. In this study, we investigated the mechanisms underlying high TSWV resistance in a specific tomato line after experimental inoculation, despite not possessing any known TSWV resistance genes. The candidate causal genes of disease resistance traits were finely mapped by constructing different genetic populations and performing bulk segregant analysis sequencing. This approach identified *SlDnaJ* (Solyc10g081220) as a key locus potentially regulating TSWV resistance. We determined a structural variant of *SlDnaJ* (designated *Sldnaj*) containing a 61-bp promoter sequence deletion that was specifically present in the germplasm of the susceptible M82 tomato plant lines. *Sldnaj*-knockout transgenic plants were significantly more resistant to TSWV than wild-type plants. Up-regulated expression of *Sldnaj* affected the salicylic acid/jasmonic acid signaling pathway, which induced and promoted the systemic infection of TSWV in M82 susceptible plants. In summary, this study identified a new candidate TSWV susceptibility gene with a natural deletion variation in tomato. These findings provide insights into the molecular mechanism underlying pathogen resistance while offering a target for breeding strategies of tomato with TSWV resistance.

## Introduction

Tomato spotted wilt virus (TSWV) is one of the 10 most destructive plant viruses recognized worldwide, infecting over 1090 plant species across 90 families [1]. The global rapid spread of the invasive thrip species *Frankliniella occidentalis* has accelerated the impact of TSWV [2], leading to serious economic losses in agronomic crops, including tomato, peppers, soybean, and peanuts [3, 4]. Tospoviruses, including TSWV, cause approximately $1 billion in global economic annual losses, posing a serious threat to global vegetable crop production [5]. As an economically important horticultural crop, tomato (*Solanum lycopersicum*) occupies an important role in the global vegetable trade. In 2022, the total tomato harvest area exceeded 4.9 million hectares, with production surpassing 186 million tons (FAOSTAT, 2023). However, TSWV systematically infects tomato plants, leading to symptoms such as dwarfing, difficulty in fruit setting, fruit shrinkage, and necrotic spots, which seriously compromise the yield and quality, posing a significant challenge to tomato production.

Currently, the most environmentally friendly and effective measure for preventing TSWV is the use of resistant cultivars. Eight resistance genes have been recognized in cultivated and wild tomatoes to date, including *Sw-1a*, *Sw-1b*, *sw-2*, *sw-3*, *sw-4*, *Sw-5*, *Sw-6*, and *Sw-7*. However, the resistance provided by *Sw-1a*, *Sw-1b*, *sw-2*, *sw-3*, and *sw-4* against specific TSWV isolates has been rapidly diminishing [6]. In addition, *Sw-6* confers a lower degree of resistance than *Sw-5*, and this situation is further exacerbated by the emergence of more aggressive TSWV isolates [7]. *Sw-7*, which has not been cloned, is located within a small interval on chromosome 12 and confers broad TSWV resistance. Despite its potential, *Sw-7* is rarely used in tomato disease resistance breeding [8]. *Sw-5*, which was cloned from *Solanum peruvianum* and located on chromosome 9, has six homologous genes (*Sw-5a*, *Sw-5b*, *Sw-5c*, *Sw-5d*, *Sw-5e*, and *Sw-5f*) [9, 10]. Among these, *Sw-5b* is the most widely and effective gene in the breeding of disease-resistant tomato [9]. However, the widespread use of cultivars harboring *Sw-5b* has caused the emergence of high concentrations of resistance-breaking TSWV isolates [11]. A novel *Sl5R-1* gene, which was fine-mapped by quantitative trait locus (QTL) analysis, was confirmed to play a key role in TSWV resistance [12]. *Sl5R-1* and *Sw-5* belong to the *NB-LRR* (*NLR*) gene family and largely confer TSWV resistance via their conserved *NB-LRR* domain [13–15]. In addition, many studies have confirmed that molecular chaperones have different defense capabilities, with some able to enhance plants disease resistance, while others having the opposite effect to ultimately enhance the plant’s susceptibility to pathogens [16–19]. Nevertheless, there is limited research on the roles of molecular chaperone genes in the regulation of TSWV resistance in tomato.

Heat shock proteins (HSPs) are the largest class of molecular chaperones, which are crucial for plants to adapt to constantly changing environmental conditions [20]. HSPs are classified into six families based on their molecular weight, including small HSP, HSP40, HSP60, HSP70, HSP90, and HSP100 [20, 21]. HSP70, also known as DnaK, is one of the most abundant members of the HSP family in plants, which plays a key role in protein quality control, requiring two co-chaperones for carrying out its function [22, 23]. HSP40, also known as DnaJ and J-protein, recognizes unfolded substrates and transfers them to DnaK, promotes the ATP hydrolysis of HSP70s, and causes conformational changes of the stable chaperone proteins [24]. DnaJ proteins are classified into four classes, including I, II, III, and DnaJ-like, based on the existence of a J domain, G/F domain, and zinc-finger structure and the absence of the His–Pro–Asp (HPD) tripeptide motif in the J domain [25]. DnaJ proteins play a vital role in biotic and abiotic stress response mechanisms. For instance, the transcription levels of all 42 putative *SlHSP20* genes identified in tomato were strongly induced by the fungal pathogens *Botrytis cinerea* and TSWV [26]. The DnaJ-like protein (LeCDJ2) cloned from tomato leaves directly interacts with HSP70 protein and participates in the defense response against pathogenic bacteria. *LeCDJ2* overexpression in tobacco significantly enhanced the plant’s resistance to *Pseudomonas solanacearum* [27]. In addition, DnaJs have been shown to participate in hormone-regulated disease resistance. For example, Arabidopsis AtJ1 is required for the abscisic acid (ABA) response and the expression of LeCDJ2 is triggered by salicylic acid (SA) [27]. However, the roles of the co-chaperone protein DnaJ in TSWV resistance in tomato remain largely unexplored.

To fill this gap, the aim of this study was to identify a new TSWV resistance gene using bulk segregant analysis sequencing (BSA-seq) and the QTL fine-mapping strategy. We found that the mapped gene *SlDnaJ* had a single base substitution in the coding sequence and a 61-bp deletion in the promoter region. This deletion variation increases the expression dosage of the variant gene (named *Sldnaj*), which appears to affect the plant's defense response to TSWV through altering the gene expression of key regulators in the SA/jasmonic acid (JA)-signaling pathway with consequent changes in the immune system of plants. These findings can provide new insights into the mechanisms contributing to TSWV resistance along with new targets for practical applications in tomato disease resistance breeding programs.

## Results

### Characteristics of TSWV infection in susceptible and resistant tomato lines

**Figure 1:**
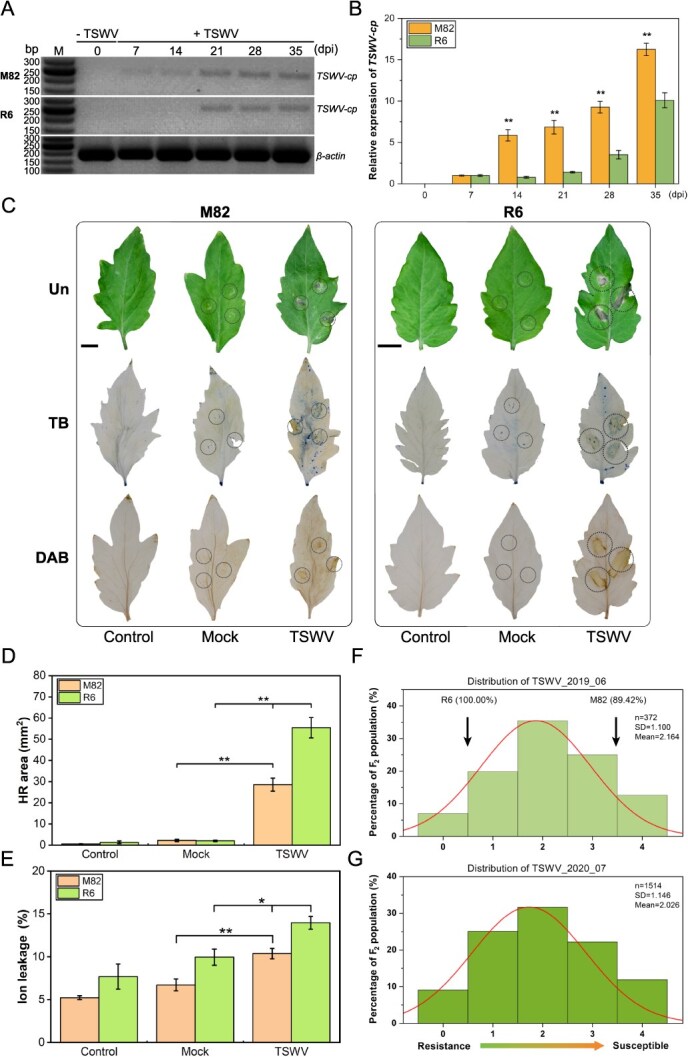
Phenotype of the susceptible parent line M82, resistant parent line R6, and their F_2_ populations after infection with TSWV. **(A)**  *TSWV-cp* was identified in M82 and R6 plants using RT-PCR at 0, 7, 14, 21, 28, and 35 days postinfection (dpi). The upper, middle, and lower electrophoresis images represent *TSWV-cp* in M82, *TSWV-cp* in R6, and *β-actin* as a standard, respectively. (**B**) *TSWV-cp* expression level in M82 and R6 plants quantified using RT-qPCR. (**C**) Hypersensitive response (HR) in TSWV-infected disease-resistant R6 plants at 7 dpi. Phenotypes were observed in unstained (Un), diaminobenzidine (DAB)-stained, and trypan blue (TB)-stained samples. Control represents the normal leaf; mock represents the phosphate buffer control. Scale bar = 10 mm. The thin dotted circle is 5 mm and the thick dotted circle is 10 mm. (**D**) Quantification of the HR (cell necrosis) response area in M82 and R6 plants. (**E**) Quantification of electrolyte leakage as a measure of ion conductivity to evaluate the HR in the leaves of M82 and R6 plants. The error bars represent the standard deviations of three biological replicates. One asterisk and two asterisks mean significant differences at *P* < 0.05 and *P* < 0.01, respectively. (**F–G**) Distribution of disease ratings in the F_2_ population inoculated with TSWV in 2019 (**F**) and 2020 (**G**). The *x*-axis shows the disease severity score, ranging from 0 to 4 (Fig. S2). The curve indicates the normal distribution.

To evaluate the resistance of tomato plants against TSWV, the parental lines M82 and R6 were artificially inoculated with the virus and evaluated at 0, 7, 14, 21, 28, and 35 days postinfection (dpi). Significant phenotypic differences were observed between the two parental lines from 14 to 35 dpi (Fig. S1). By 35 dpi, the susceptible M82 plants exhibited typical TSWV symptoms such as leaf chlorosis, purple veins, necrotic lesions, and plant stunting. In contrast, R6 plants displayed high resistance to TSWV, exhibiting no such symptoms. Further identification by PCR revealed that R6 plants did not contain *Sw-5* and *Sw-7* genes [12]. RT-PCR and RT-qPCR demonstrated that the *TSWV-cp* expression level in M82 plants peaked from 14 to 35 dpi and was significantly higher than that of R6 plants (Fig. 1A and B). Diaminobenzidine (DAB) and trypan blue (TB) staining indicated that the resistant R6 plants had a significantly greater hypersensitive response (HR), as manifested by a cell necrosis reaction and H_2_O_2_ accumulation compared to those of the susceptible M82 plants at 7 dpi. The R6 plants showed an intense necrosis reaction, with inoculated leaves exhibiting strong curling, whereas M82 plants exhibited only weak necrosis at the inoculation site (Fig. 1C). Image J analysis confirmed that R6 plants produced a significantly larger necrotic area compared to that formed in M82 plants (Fig. 1D). This greater extent of necrosis was further corroborated by the increased level of ion leakage in R6 plants (Fig. 1E). In summary, resistant R6 plants showed a strong HR and extensive H_2_O_2_ accumulation upon TSWV infection, indicating R6 as a novel tomato germplasm with high resistance to TSWV.

### Resistance to TSWV is controlled by a single dominant locus

To study the inheritance of the TSWV resistance trait in tomatoes, we constructed F_2_ populations using the resistance genotype R6 (parent P1) and the susceptible genotype M82 (parent P2). Six generations of the population were inoculated with a locally isolated TSWV strain to assess the performance characteristics of individuals plants. The TSWV disease severity score showed phenotypic variation within the F_2_ populations, ranging from 0 to 4, in both 2019 and 2020 (Fig. 1F and G, Fig. S2). Phenotype analysis showed that the data followed a continuous normal distribution curve with the traits exhibiting transgressive segregation (Fig. 1F and G). The Shapiro–Wilk test confirmed that the phenotype data of multiple environments were regularly distributed in different seasons (Table 1). Analysis of the resistance traits of F_2_ populations showed a ratio of resistance-to-susceptible plants of 275:97 and 1118:396 in 2019 and 2020, respectively, which is consistent with a 3:1 segregation pattern (χ^2^ = 0.229 and 1.079, respectively; both *P* > 0.05; Table 1), indicating that the resistance trait follows a pattern of single dominant inheritance. To further validate this finding, we constructed a BC_1_P2 population using F_1_ and M82 plants. The progeny showed a resistance to susceptible ratio of 41:37, conforming to a 1:1 segregation ratio (χ^2^ = 0.205, *P* > 0.05; Table 1). Additionally, all progeny in the BC_1_P1 population were resistant to TSWV, further confirming that the resistance trait is regulated by a single dominant locus.

**Table 1:** Segregation of the TSWV-susceptible and -resistant trait in the populations of the resistant parent (P_1_) and susceptible parent (P_2_)

Generation	Total No. of plants	Segregation		Expected segregation	χ2 value	*P*-value	Significance
		^a^Resistance	^a^Susceptible				
P1(R6)	46	46	0	–	–	–	–
P2 (M82)	63	0	63	–	–	–	–
F_1_	55	55	0	–	–	–	–
BC_1_P1	54	54	0	–	–	–	–
BC_1_P2	78	41	37	1:1	0.2051	0.6506	*P* > 0.05
^b^F_2_–2019	372	275	97	3:1	0.2294	0.6320	*P* > 0.05
^b^F_2_–2020	1514	1118	396	3:1	1.0788	0.2990	*P* > 0.05

a Disease ratings 0, 1 and 2 were defined as resistance; disease ratings 3 and 4 were defined as susceptible. Disease ratings were scored on a scale of 0 to 4 disease intensity, consisting of asymptomatic, mild necrosis, moderate necrosis, severe necrosis, and necrosis of the entire plant, respectively.

b F_2_ population established for 2 years from the population of TSWV_2019_06 and TSWV_2020_07.

### Identification of a candidate QTL for TSWV resistance

**Figure 2:**
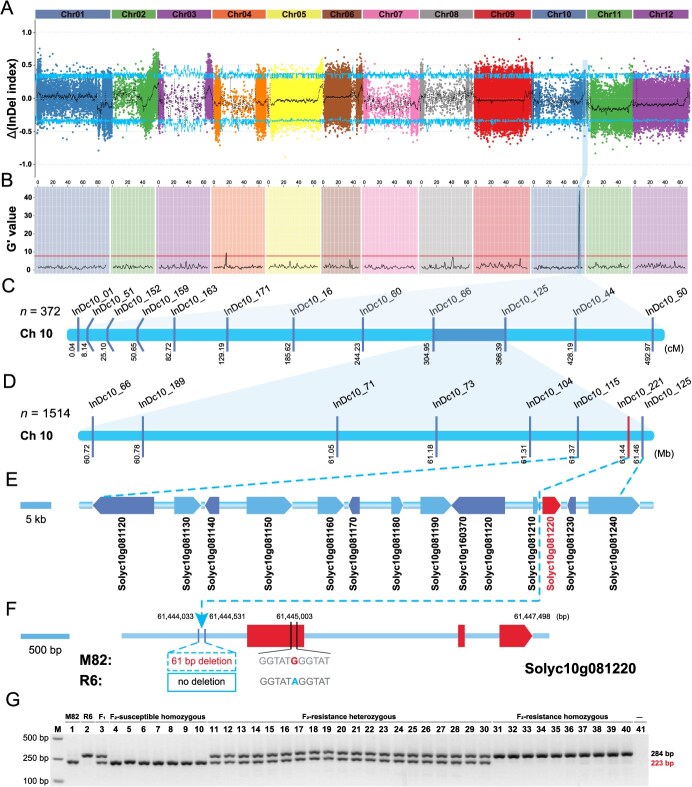
Identification of *SlDnaJ* (*Solyc10g081220*) as a candidate gene that confers resistance to TSWV in tomatoes based on fine mapping. (**A**) The ∆(InDel index) derived from BSA-seq analysis. The x-axis represents the 12 tomato chromosomes. The central line represents the fitted ∆InDel index. The upper and lower lines indicate a confidence level of 95% as the threshold for screening. (**B**) Identification of quantitative trait loci (QTLs) for TSWV resistance using QTLseqr. The line indicates the threshold associated with the resistance trait at the 95% confidence interval. (**C**) Initial mapping of *SlDnaJ* on chromosome 10 using 372 individuals of the F_2_ population (TSWV_2019_06). (**D**) Fine mapping of *SlDnaJ* to estimate its location in an 85.45-kb region between markers InDc10_115 and InDc10_125 using 1514 individuals of the F_2_ population (TSWV_2020_07). (**E**) Annotated models in the fine-mapped region based on the Heinz 1706 (SL4.0 genome version and ITAG4.1 annotation). (**F**) Gene structure model of *SlDnaJ*. The 61-bp deletion in the promoter and the single base substitution in the coding region of the *SlDnaJ* are marked. (**G**) PCR results for different plant populations using the InDc10_221 marker developed with the 61-bp deletion (as shown in D). M indicates the DL2,000 DNA marker.

To identify candidate QTLs responsible for TSWV resistance, we used the QTL-seq approach with the F_2_ populations. We selected genomic DNA of 30 highly susceptible and 30 highly resistant F_2_ individuals, combining them into the TwS04_bulk and TwR03_bulk pools, respectively. The four pools, TwR01 (R6), TwS02 (M82), TwS04_bulk, and TwR03_bulk, were sequenced using the Illumina NovaSeq 6000 platform, producing a total of 79.45 G clean reads (Table S1). Specifically, 18.69 Gb clean reads were generated for the two inbred lines TwR01 and TwS02 (10× genome coverage), while 60.82 Gb data were generated for TwR03_bulk and TwS04_bulk from F_2_ populations (30× average genome coverage) with high quality (Q20 ≥ 97.52%, Q30 ≥ 92.96%; Table S1). The mapping rates of the four pools ranged from 97.07% to 99.32%, with average depths of 10.15× for TwR01, 99.42× for TwS02, 30.47× for TwR03_bulk, and 32.32× for TwS04_bulk (Table S2). The insertion–deletion (InDel) index was then calculated by subtracting the InDel index value from the TwR03_bulk and TwS04_bulk (Fig. 2A, Fig. S3). Under the null hypothesis, candidate regions above the threshold were selected as the regions harboring major QTLs for the resistance trait. At the 95% significance level, we identified seven candidate QTLs (Table S3). Using QTLeqr, the G′ value was mapped to the genome position. At the 99% significance level, the G′ value for one genomic region (SL4.0ch10: 59751511-61 453 256) significantly exceeded the threshold (G′ value = 43.77; Fig. 2B, Table S4). This region ‌examined in the QTL-seq analysis was then designated as the target region harboring a variation as the candidate cause of the resistance trait.

### Identification of a large sequence deletion in the *SlDnaJ* promoter

To fine map the genomic region associated with TSWV resistance, we performed linkage analysis using the F_2_ populations. A significant signal located between 59.75 Mb and 61.45 Mb on chromosome 10 was identified (Fig. 2A and B). We used the genotyping data of 12 polymorphic InDel markers (Table S5) and the disease ratings data of individual plants in the F_2_ population (TSWV_2019_06) to construct the genetic map, which narrowed the region to a 1.70-Mb interval between markers InDc10_66 and InDc10_125 (Fig. 2C). To further fine map the region, we developed 36 InDel markers; after quality control, linkage analysis was ultimately performed using the genotyping data of eight valid InDel markers (Table S6) and the phenotypic data of 1514 individuals (TSWV_2020_07). This analysis further narrowed down the resistance QTL to the region between InDc10_115 and InDc10_125 (SL4.0ch10: 61371874–61 457 328), which spans approximately 85.45 kb and harbors 14 genes (Fig. 2D and E; Table S7). Within this region, we identified a 61-bp deletion (SL4.0ch010: 61443987–61 444 048) located 639-bp upstream of the gene Solyc10g081220 (Fig. 2F). This gene, designated *SlDnaJ*, belongs to the HSP family and encodes a DnaJ chaperone protein. In addition, we found a non-synonymous variation (G/A) between M82 and R6 at position 61 445 003, which results in a mutant base of A in R6. The marker InDc10_221 was then designed to target the 61-bp deletion, which ‌exhibited complete co-segregation with the phenotype of F_2_ individuals (Fig. 2G).

### Conservation of the deleted promoter sequence and substituted base in *SlDnaJ*

To assess the conservation of the 61-bp promoter deletion and single-base substitution, we sequenced the 1-kb promoter sequence and the coding sequence (CDS) of *SlDnaJ* in susceptible M82 plants. We found that the substituted base in the CDS of *SlDnaJ* was strongly conserved in resistant and susceptible plants, with ‘A’ at this position in the resistant plants R6, F_1_, and F_2_-homozygous lines, and ‘G’ at this position in susceptible plants M82, Heinz 1706, Moneymaker (MM), Alisa Craig (AC), and F_2_-homozygous lines (Fig. S7A). The single-base G-to-A substitution in *SlDnaJ* leads to the conversion of arginine (R) to glycine (G) at the N-terminal region of the encoded protein (Fig. S5A). Three-dimensional structure analysis of SlDnaJ/Sldnaj proteins showed no difference expect at the site of the amino acid substitution from R to G (Fig. S6A and B). In contrast, the 61-bp region of the *SlDnaJ* promoter sequence was not missing in the tomato lines R6, MM, AC, R7, H8, H19, and H149, demonstrating that this deletion is highly specific to the M82 line (Fig. S4). Thus, the 61-bp deletion in the promoter sequence of *SlDnaJ* occurs only in M82, whereas the base substitution (G) is highly conserved among susceptible plant lines. In addition to the deletion of 61 bp in the promoter region, additional variants were identified between M82 and R6 plants, including single base deletions, substitutions, and insertions (Fig. S5B).

The detection of cis-acting regulatory elements revealed the presence of SA- and JA-responsive elements within the *SlDnaJ* promoter region. Additionally, the promoter from R6 contained TC-rich repeats and TCA elements, with one more CAAT-box and TATA-box (a core promoter element) identified in the promoter of M82 compared to that of R6 (Fig. S7C). In particular, the 61 bp sequence deleted in M82 but not in R6 contained a TC-rich repeat and a TATA-box element. Further analysis of transcription factor-binding sites revealed that the *SlDnaJ* promoter in R6 had five more ethylene-responsive factor and bZIP elements than found in M82 (Fig. S7C). However, there were 11 more DNA-binding with one finger elements in M82 than in R6 (Fig. S7D). In particular, the 61-bp sequence deleted in M82 contained an E2F/DP transcription factor binding motif in R6. RT-qPCR demonstrated that *SlDnaJ* expression was significantly up-regulated in M82 at 14 dpi and 21 dpi, followed by a significant decrease in the expression level at 28 dpi compared to the measured expression levels in R6 at the same time points (Fig. S7B). Crucially, the expression level of *SlDnaJ* was consistently significantly lower in R6 plants than in M82 plants. Collectively, these findings suggested that the difference of motifs in the amino acid sequences be a factor contributing to the up-regulation of *SldnaJ* expression in M82.

### Transient overexpression of 35S::*SlDnaJ* from R6 plants induces the HR to TSWV

To confirm the resistance function of *SlDnaJ*, the CDS in the two parent plants was cloned by PCR, which was used for cluster analysis and transient overexpression verification. *SlDnaJ* was identified as a characteristic *HSP40* gene, including a conserved DnaJ domain and transmembrane domain. The neighbor-joining phylogenetic tree of 73 DnaJ proteins from 54 species showed that the homologous DnaJ proteins of Solanaceae plants such as the tomato species *Solanum pennellii* and *Solanum verrucosum* were in the same evolutionary branch, indicating that they may have a common origin, with the closest genetic relationship found between SlDnaJ and SpeDnaJ in *Solanum pennellii* (Fig. S8).

**Figure 3:**
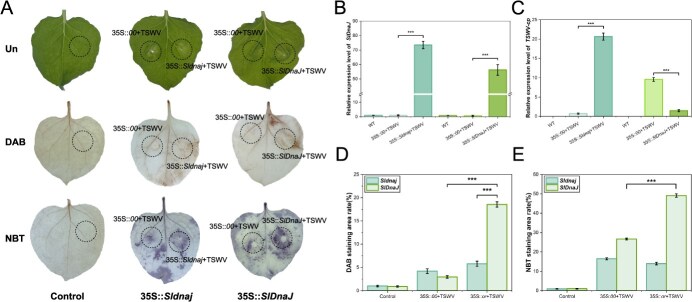
Hypersensitive response (HR) triggered by transient overexpression of 35S::*SlDnaJ*. **(A)** HR triggered by transient overexpression of 35S::*SlDnaJ/Sldnaj* in tobacco at 7 days postinoculation (dpi), visualized by observations of unstained (Un), DAB-stained, and nitroblue tetrazolium (NBT)-stained sections. The control represents the normal leaf tissue. **(B)** RT-qPCR analysis of *SlDnaJ* and *Sldnaj* transcript levels in 35S::*SlDnaJ/Sldnaj* transiently overexpressed plants at 7 dpi. **(C)** RT-qPCR analysis of *TSWV-cp* transcript levels in 35S::*SlDnaJ/Sldnaj* transiently overexpressed plants at 7 dpi. **(D)** Quantification of the DAB-staining area indicating the HR in 35S::*SlDnaJ/Sldnaj* transiently overexpressed plants at 7 dpi. **(E)** Quantification of the NBT staining area indicating the HR in 35S::*SlDnaJ/Sldnaj* transiently overexpressed plants at 7 dpi. The error bars represent the standard deviations of three biological replicates. Three asterisks mean significant differences at *P* < 0.001.

Detection of green fluorescent protein (GFP) signals demonstrated that SlDnaJ protein was localized in the cytomembrane and nucleus, whereas Sldnaj protein exhibited a stronger signal in the cytomembrane (Fig. S9), suggesting that the transmembrane domain is required for cytomembrane localization. Transient expression of *SlDnaJ* in *Nicotiana benthamiana* triggered HR-mediated cell necrosis and H_2_O_2_ accumulation in response to TSWV infection, which was not found with transient expression of the variant *Sldnaj* (Fig. 3A). The *SlDnaJ/ Sldnaj* transcript levels were significantly higher in tobacco with transient expression of 35S::*SlDnaJ/Sldnaj* at 7 dpi than those in the WT and control plants (Fig. 3B), confirming that *SlDnaJ/Sldnaj* was successfully overexpressed in tobacco leaves. The *TSWV-cp* expression level in the *SlDnaJ/Sldnaj*-transformed plants was significantly greater than that in the WT and control plants at 7 dpi (Fig. 3C), confirming that expression of these genes suppressed the resistance to TSWV. DAB and NBT staining experiments showed extremely high necrotic areas, demonstrating a strong HR (Fig. 3D and E). The above experimental results suggested that *SlDnaJ* induces an HR as a regulation mechanism of the TSWV resistance response.

### *SlDnaJ* silencing disrupts TSWV resistance in R6

**Figure 4:**
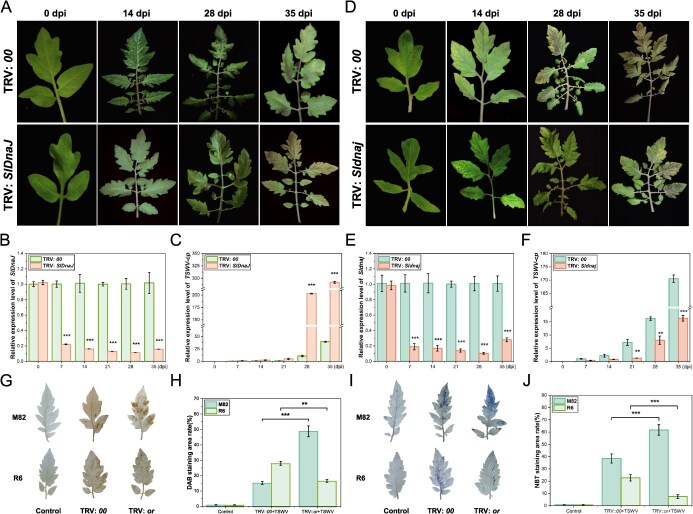
Response of resistant plants to virus-induced gene silencing (VIGS) of *SlDnaJ*/*Sldnaj* after TSWV inoculation. **(A)** Phenotype of *SlDnaJ*-silenced R6 resistant plants at 0, 14, 28, and 35 days postinoculation (dpi) of TSWV. **(B, E)** RT-qPCR analysis to confirm the silencing efficiency for *SlDnaJ/Sldnaj* expression in *SlDnaJ/Sldnaj*-silenced plants at 0, 7, 14, 21, 28, and 35 dpi. **(C, F)** RT-qPCR detection of *TSWV-cp* expression in *SlDnaJ/Sldnaj*-silenced plants at different periods (0, 7, 14, 21, 28, and 35 dpi). Gene transcript levels were normalized to the level of *SIEF1-α-actin*. **(D)** Phenotype of *Sldnaj*-silenced susceptible plants M82 at 0, 14, 28, and 35 dpi. **(G)** DAB staining of *SlDnaJ/Sldnaj*-silenced plants at 7 dpi. **(H)** DAB staining of *SlDnaJ/Sldnaj*-silenced plants. **(I)** NBT staining of *SlDnaJ/Sldnaj*-silenced plants at 7dpi. **(J)** NBT staining area of *SlDnaJ/Sldnaj*-silenced plants. The error bars represent the standard deviations of three biological replicates. Two asterisks and three asterisks mean significant differences at *P* < 0.01 and *P* < 0.001, respectively.

To investigate the role of *SlDnaJ* in TSWV resistance, we performed virus-induced gene silencing (VIGS) using Tobacco rattle virus (TRV):*SlDnaJ* and TRV:*Sldnaj* vectors in the resistant R6 plants and susceptible M82 plant. Silencing *SlDnaJ* in R6 led to the significant down-regulation of *SlDnaJ* expression (Fig. 4B). This in turn resulted in enhanced leaf symptoms and reduced resistance upon TSWV inoculation (Fig. 4A), as well as significant down-regulation of *TSWV-cp* expression at 28 and 35 dpi (Fig. 4C). Conversely, silencing and down-regulation of *Sldnaj* expression in the susceptible M82 plants (Fig. 4E) alleviated leaf symptoms and enhanced resistance (Fig. 4D), with significant up-regulation of *TSWV-cp* expression at 21 to 35 dpi compared to that of control plants (Fig. 4F). *Sldnaj*-silenced plants exhibited deeper DAB and NBT staining, with a nearly three times increase in the DAB staining area and ~ 20% increase in the NBT staining area (Fig. 4G and H). In contrast, *SlDnaJ*-silenced plants showed lighter staining, with a 12% and 14% reduction in the DAB and NBT staining area, respectively (Fig. 4I and J). These findings further supported *Sldnaj* as a key gene regulating TSWV infection in susceptible tomato plants.

### Overexpression of *SlDnaJ* enhances TSWV resistance in susceptible plants

**Figure 5:**
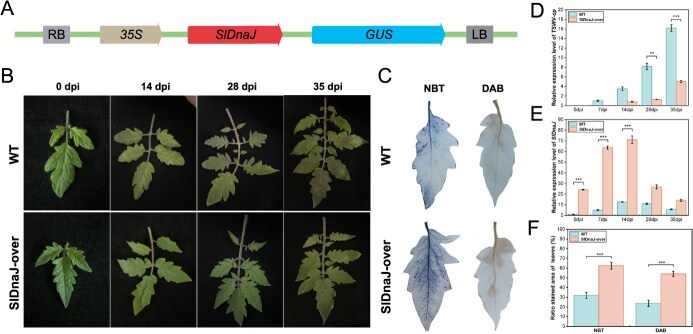
Overexpression of *SlDnaJ* in the susceptible tomato plant M82 enhanced resistance to TSWV. (**A**) Schematic diagram showing construction of the *SlDnaJ* overexpression vector. The wild-type (WT) sequences and overexpression line (*SlDnaJ*-over) were identified by PCR of the *SlDnaJ* CDS region from T_1_ plants in the M82 background. (**B**) Representative leaf disease phenotypes of the WT and *SlDnaJ*-over line at 0, 14, 28, and 35 days postinoculation (dpi) of TSWV. (**C**) DAB and NBT staining of the *SlDnaJ*-over line at 7 dpi. (**D**) RT-qPCR analysis of *TSWV-cp* expression in the *SlDnaJ*-over line at 0, 7, 14, 28, and 35 dpi. (**E**) RT-qPCR analysis of the *SlDnaJ* transcript level in the *SlDnaJ*-over line at 0, 7, 14, 28, and 35 dpi. (**F**) The ratio of DAB and NBT staining areas in the *SlDnaJ*-over line. The error bars represent the standard deviations of three biological replicates. Two asterisks and three asterisks indicate significant differences at *P* < 0.01 and *P* < 0.001, respectively.

To verify that *SlDnaJ* confers resistance to TSWV, we generated transgenic M82 plants overexpressing *SlDnaJ* driven by the 35S promoter (Fig. 5A). PCR confirmed the presence of the *SlDnaJ* CDS in T_1_ plants. Compared to those of the WT plants, the transgenic *SlDnaJ*-overexpressing (*SlDnaJ*-over) lines exhibited significantly milder leaf symptoms (Fig. 5B). In the *SlDnaJ*-over line, *SlDnaJ* expression was significantly up-regulated at 7–28 dpi (Fig. 5E), while *TSWV-cp* expression was significantly down-regulated from 14 to 35 dpi compared to those of WT plants (Fig. 5D). DAB and NBT staining revealed increased staining intensity in the *SlDnaJ*-over line (Fig. 5C), with the staining area nearly doubling (Fig. 5F).

Further analysis of antioxidant enzyme activity, phytohormone content, and the expression levels of key SA and JA signaling pathway regulators showed that SA and JA accumulation was significantly greater in the *SlDnaJ*-over line compared to that of WT plants (Fig. S10A and B). The activities of antioxidant-related enzymes, including peroxidase (POD), superoxide dismutase (SOD), and catalase (CAT), were also significantly enhanced in the overexpression line (Fig. S11A–C). Specifically, the expression levels of SA/JA signaling pathway regulator genes and antioxidant-related enzyme activities were higher in the *SlDnaJ*-over line than in WT plants from 7 to 35 dpi (Fig. S11D–F; Fig. S12). These results further supported that *SlDnaJ* regulates the resistance to TSWV in tomato.

### Gene editing of *Sldnaj* enhances resistance to TSWV in susceptible plants

**Figure 6:**
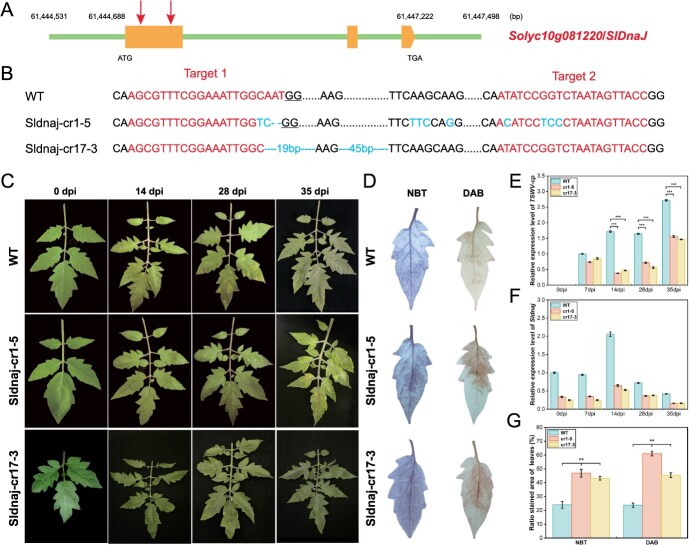
The characteristics of CRISPR/Cas9-edited *Sldnaj* lines after TSWV inoculation. (**A**) Diagrammatic sketch of *SlDnaJ* targeting the two single-guide RNAs (sgRNAs). (**B**) Sequences of the edited regions of the CRISPR/Cas9-edited *Sldnaj* mutants (*Sldnaj*-cr1–5 and *Sldnaj*-cr17–3) and wild-type (WT). The two sgRNA target sites are indicated in Target 1 and Target 2 and the altered sequences in the edited line are indicated in blue. The *Sldnaj*-cr alleles were confirmed by sequencing of the PCR products from two T_1_ plants in the M82 background. (**C**) The leaf symptom phenotypes of the WT, *Sldnaj*-cr1–5, and *Sldnaj*-cr17–3 lines at 0, 14, 28, and 35 days postinoculation (dpi). (**D**) DAB and NBT staining of *Sldnaj*-cr lines at 7 dpi. (**E**) RT-qPCR detection of the expression of *TSWV-cp* in *Sldnaj*-cr lines at 0, 7, 14, 28, and 35 dpi. (**F**) RT-qPCR detection of the *Sldnaj* transcription levels in the *Sldnaj*-cr lines at 0, 7, 14, 28, and 35 dpi. (**G**) The ratio of DAB and NBT staining areas of the *Sldnaj*-cr lines. The error bars represent the standard deviations three biological replicates. Two asterisks and three asterisks indicate significant differences at *P* < 0.01 and *P* < 0.001, respectively.

To further validate *SlDnaJ* as a causative gene underlying the *Sldnaj* genotype, we employed the CRISPR/Cas9 system to ‌create‌ knock-out mutation using two single-guide RNAs designed to target the first exon of *SlDnaJ* (Fig. 6A). These gene-edited plants were verified by sequencing of the PCR products (Fig. 6B). We evaluated the second-generation (T_1_) diploid lines homozygous for the edited mutant alleles, and found that the leaf symptoms in the *Sldnaj*-cr mutants (cr1–5 and cr17–3) were significantly weaker than those in WT plants (Fig. 6C). In these *Sldnaj*-cr lines, the *Sldnaj* expression was considerably down-regulated at 7 and 35 dpi (Fig. 6F), and the *TSWV-cp* expression level was significantly reduced compared with that of the WT at 14–35 dpi (Fig. 6E). DAB and NBT staining analysis showed increased staining intensity in the *Sldnaj*-cr lines (Fig. 6D), with the NBT and DAB staining area increasing by more than 2- and 3-fold, respectively (Fig. 6G).

Analysis of the phytohormone content and activities of antioxidant enzymes indicated significantly higher SA accumulation in the *Sldnaj*-cr line compared with that of WT plants at 14 and 28 dpi (Fig. S13A), along with higher JA accumulation at 35 dpi (Fig. S13B). The enzyme activities of POD, SOD, and CAT were markedly enhanced in the gene-edited line at 7 to 35 dpi (Fig. S14A–C). The expression levels of key genes of the SA/JA signaling pathway and the activities of antioxidant-related enzymes were all higher in the *Sldnaj*-cr line than those in WT plants at 7 to 35 dpi (Fig. S14D–F; Fig. S15A, B, H, and I). These results indicated that *Sldnaj* plays an essential‌ role in controlling TSWV infection in susceptible tomato plants.

## Discussion

Viral diseases seriously endanger the production and quality of crops worldwide, especially vegetable crops such as tomatoes and peppers. TSWV is an RNA virus that markedly impacts the growth of tomato plants, and high variability of the virus has resulted in the loss of resistance conferred by the application of resistance genes in molecular breeding [11, 28]. The successful infection of pathogens requires the use of plant disease-susceptible genes. Mutations in disease-susceptible genes usually confers broad-spectrum and long-lasting disease resistance to plants [29, 30]. In this study, we identified *Sldnaj* as a candidate susceptibility gene in tomato, providing evidence for its crucial role in the regulation of TSWV resistance.

### Resistance to TSWV is a stable trait phenotype

In QTL mapping for disease resistance, the data of the resistance phenotype provide key information for accurate positioning, which can further provide insight into the environmental effects on resistant and susceptible phenotypes. In this study, M82 and R6 were identified as TSWV-resistant and -susceptible parent tomato lines, respectively, with extremely significant differences in the response to infection. Phenotypic data with respect to TSWV resistance of the sixth-generation populations were obtained by growing the plants under green greenhouse conditions for 2 years (in 2019 and 2020). The phenotype observations confirmed that the 2-year F_2_ population showed high phenotypic variation to TSWV and was relatively stable. This conclusion is consistent with previous studies [12]; however, a previous evaluation of disease resistance was carried out in tomato under field conditions and disease resistance was evaluated based on a scale ranging from 1 to 10 scales in a study with peanuts [31]. Therefore, the present study provides a new foundation for the precise mapping of TSWV resistance QTLs.

### Fine mapping of *SlDnaJ*

The combination of a next-generation sequencing gene mapping strategy and a fine-mapping toolkit is an effective approach to mine target genes for crop traits. Presumably, the combination of statistical methods can accurately locate the interval and reduce the workload. In this study, we found that the R6 tomato plant line demonstrated stable, high resistance to TSWV, and resistance was a dominant trait without the presence of the known *Sw-5* and *Sw-7* resistance genes [12]. Genetic analysis confirmed that the inheritance pattern conforms to Mendel’s segregation law, demonstrating that the resistance trait is regulated by a single dominant locus. However, association analysis using the SNP-index and InDel-index algorithms in QTL-seq identified seven QTLs at the 95% significance level, spanning a very large region on chromosome 10 in tomato. Association analysis using the G′ value method of the QTLseqr package identified a genomic region that significantly exceeded the threshold at the 99% significance level. Therefore, the interval was finally narrowed to position 61 059001-61453256 through the combination of the two identified interval areas. We further developed polymorphic InDel markers for this region, and used F_2_ populations (TSWV_2019_06: 372, and TSWV_2020_07: 1514 individuals) for fine mapping to narrow the region between InDc10_115 and InDc10_125, which harbored 14 genes in an approximately 85.45-kb region. Similarly, QTLseqr and calculated G′ values were plotted to a genomic region associated with watermelon rind hardness at a 99% significance level [32]. In this study, we found that among the 14 genes in this region, only *SlDnaJ*(Solyc10g081220) had a 61-bp deletion and a non-synonymous substitution (G/A), and was associated with plant defense capabilities. Therefore, *SlDnaJ* was considered the best candidate gene associated with resistance within the mapped region. Protein domain analysis showed that SlDnaJ had a conserved DnaJ domain, indicating a typical type III HSP40/DnaJ protein. DnaJ family members play a key role in assisting viral entry into the nucleus, participating in viral replication, and helping the correct folding of viral proteins [33, 34]. Thus, *SlDnaJ* is considered to be a candidate gene locus regulating TSWV resistance in tomato.

### Downregulation of *SlDnaJ* expression confers resistance to TSWV

Two differential sites were found in the promoter regions and CDS of *SlDnaJ* between M82 and R6 plants. The mutated base (G/A) in the CDS was strongly conserved in the resistant R6 line, causing an amino acid to change from R to G. The three-dimensional structure of the SlDnaJ and mutated Sldnaj proteins only differed at the substituted amino acid site. Furthermore, in the amino acid-based specific structure, the side chain of Arg is positively charged, while Gly is not charged. This change seems to affect the interaction between proteins and other molecules, and may also be one of the reasons for the observed difference in the disease resistance function of *SlDnaJ*. We further focused on the promoter region of *SlDnaJ*. A 61-bp deletion of the promoter was found in M82 cultivated tomato plants, showing a high degree of specificity. This deletion is not present in the *SlDnaJ* of R6 wild tomato plants. In addition, there was no deletion in Heinz 1706, Moneymaker, and Alisa Craig plants. Therefore, we speculate that the long-term artificial cultivation of cultivated tomato M82 tomato plants may have led to the deletion of 61 bp in the promoter region, which makes this cultivar susceptible to viral infection. RT-qPCR revealed that the expression level of *Sldnaj* significantly increased in susceptible M82 plants at 14 and 21 dpi, while there was no significant change in resistant R6 plants. In addition, the transcription factor-binding site and cis-acting regulatory elements analysis exhibited that the 61-bp region that is not deleted in R6 contained TC-rich repeats, a TATA-box element, and an E2F/DP transcription factor-binding motif. Both TC-rich repeats and TATA-box element are related to the defense/stress-response. The transcription factor E2F/DP and plant hormones, such as SA play important regulatory functions in plant stress [35]. Collectively, this evidence confirmed that *SlDnaJ* confers resistance to TSWV via suppressed promoter activity.

### The susceptibility gene *Sldnaj* regulates TSWV infection

A durable plant-to-pathogen immune strategy has emerged as an alternative approach for disease resistance breeding by manipulating susceptibility (*S*) genes. Several *S* genes have been excavated in important crops to date, including *Mlo* and *TaPsIPK1* in wheat and *SWEET* genes in rice [29, 30, 36, 37]; however, there are few reports of *S* genes in tomato. We found that *Sldnaj* expression was significantly up-regulated in susceptible M82 plants infected with TSWV. Therefore, we speculated that this gene may be a susceptibility gene that assists TSWV-infected plants. VIGS experiments confirmed that silencing *Sldnaj* enhanced the resistance of susceptible M82 plants. Gene editing experiments further confirmed that editing *Sldnaj* in the susceptible M82 plants weakened leaf symptoms and downregulated *TSWV-cp* expression compared to those of the WT. Enzyme activities, SA and JA accumulation, and SA/JA signaling pathway regulators showed significant differences between gene-edited and WT plants. Furthermore, *SlDnaJ* overexpression in the susceptible M82 plants alleviated the symptoms of infection, resulting in relatively mild disease resistance compared to the WT. These results all support *Sldnaj* as a TSWV susceptibility gene, suggesting its important role in virus infection of susceptible tomato plants.

Similarly, the bacterial blight resistance gene *xa13* and the susceptibility gene *Xa13* in rice only differ in the promoter region; the *Xa13* promoter is transcribed after interacting with a particular transcription activator-like effector (TALE) protein, while the *xa13* promoter mutation prevents TALE binding, resulting in resistance [38, 39]. In susceptible rice, the TALE AvrXa2-binding element is absent in the *xa23* allele promoter, resulting in the resistance protein XA23 not being expressed, leading to susceptibility [40]. Plant viruses recruit HSP70 by interacting with DnaJ proteins to promote the function of motor proteins [41]. DnaJ family proteins in tobacco were confirmed to interact with the nonstructural protein NSm of TSWV, implicating an HSP70-dependent mechanism during TSWV movement [42]. HSP70 may be recruited to the TSWV transport complex by DnaJ protein interacting with NSm. The interaction between NSm and plant DnaJ/DnaJ-like protein links the virus to the intercellular movement mechanism mediated by the plant plasmodesmata, thereby promoting the prevalence of viruses in host plants, including tobacco, tomatoes, and *Arabidopsis* [43]. A previous study indicated that the NSs silencing suppressor protein of TSWV preferentially interacts with plant defense-related proteins, including HSP90 and HSP70, carbonic anhydrase, importin, and calmodulin in tobacco, tomatoes, and *Arabidopsis* [16]. Overexpression of the molecular co-chaperone *NbSGT1* in *Nicotiana benthamiana* sped up the invasion rate of TSWV, while silencing *NbSGT1* strongly inhibited the intercellular movement of NSm, as well as systemic and local infections [44]. Therefore, in light of this previous work and our present data, it is likely that the up-regulated expression of *Sldnaj* in susceptible M82 enables the binding to more NSm proteins, and the consequent interaction between HSP70 and Sldnaj facilitates TSWV infection, resulting in the susceptible plant phenotype.

However, whether an amino acid difference between SlDnaJ and Sldnaj causes a significant difference in disease resistance and susceptibility, and the real reason for the observed up-regulation of *Sldnaj*, we plan to confirm this excellent hypothesis by designing focused experiments in our subsequent study. Although these inferences and the detailed mechanism need to be verified by subsequent experiments, *Sldnaj* is identified as a candidate susceptibility gene of great significance. Future research using advanced gene-editing technology is planned to edit the susceptible gene *Sldnaj* and to elucidate the Sldnaj protein and TSWV interaction networks, which will help to confirm the resistance mechanism and provide practical guidance to cultivate new tomato varieties with persistence and broad-spectrum resistance to TSWV.

## Materials and methods

### Plant materials and breeding strategy

Seven homozygous tomato lines, *S. lycopersicum* cv. M82, *S. lycopersicum* var. *cerasiforme* Alef. cultivar R6, Moneymaker (MM), Alisa Craig (AC), R7, H8, H19, and H149, were used in this study. The female parent M82 is a susceptible cultivar, whereas the male parent R6 exhibits high resistance to TSWV but does not carry the *Sw-5* and *Sw-7* resistance genes [12]. The two parents were crossed to produce the F_1_ generation, followed by selfing of the F_1_ plants to obtain the F_2_ population consisting of 1886 individuals. The BC_1_P1 and BC_1_P2 populations were then established by backcrossing the F_1_ plants with P1(R6) and P2(M82) plants. Phenotyping of the F_2_ population for TSWV resistance was conducted under two-years field conditions in 2019 and 2020, with planting dates at the end of March each year. A randomized complete-block design was used. All plants were cultivated under greenhouse conditions at Northwest A&F University, Shaanxi, China.

### TSWV artificial inoculation and resistance assessment

Leaf tissues with systemic infection of the TSWV isolate [12] were assayed for the presence of TSWV using the TSWV-*cp* primer (Table S8). The artificial inoculation of the TSWV isolate was based on a previously reported method [3]. The seedlings of R6, M82, BC_1_F_1_, BC_2_F_1_, F_1_, F_2_, and transgenic plants were inoculated at the three-to-four leaf stage. The inoculation assays were conducted as described previously [12]. The inoculated plants were cultured in a greenhouse with a temperature of 25°C day/18°C night and relative humidity of 60% day/95% night to monitor symptom expression. Disease intensity was evaluated according to the detection of symptoms of plants weekly, and the disease index and incidence rate were calculated as described previously [12].

### DNA extraction and BSA-seq

To rapidly identify the variation conferring the resistance phenotype in R6 plants, we used MutMap on the basis of whole-genome BSA-seq of the F_2_ separated population. Total DNA was separated from young fresh leaves using Plant DNAzol Reagent (Invitrogen). TwR01 (R6) and TwS02 (M82) were the two parents. TwR03_bulk and TwS04_bulk were respectively constructed by evenly mixing 30 highly resistant and 30 highly susceptible F_2_ individuals. The paired-end 150-bp reads were generated with a DNA fragment of approximately 350 bp. The libraries were sequenced using the Illumina NovaSeq 6000 platform. The sequencing depth of the parents had 10-fold coverage and the two mixed pools had 30-fold coverage. The paired-end reads of TwR01, TwS02, TwR03_bulk, and TwS04_bulk were mapped to the reference genome of tomato Heinz 1706 (SL4.0 build). The raw sequencing data were filtered for quality and the high-quality data were analyzed by BLAST. Burrows–Wheeler Aligner software was used to compare the data to the tomato genome to detect the deep sequencing, gene coverage, and gene variation. Genome Analysis ToolKit software was used to retrieve SNPs and InDels in four sequencing libraries. Correlation analysis was performed using Euclidean distance, SNP-index, and InDel-index algorithms. The G′ value was analyzed with the QTLseqr R package [45].

### Molecular marker development and fine mapping of target genes

The whole-genome resequencing data of TwR01, TwS02, TwR03_bulk, and TwS04_bulk were compared to identify InDel variations to develop InDel markers. A total of 36 InDels showing strong associations were selected for InDel markers development. Among these, 19 different markers between the susceptible and resistant plants were used for fine location (Table S5; Table S6). These InDel markers were used for genotyping by PCR, and the phenotypes of individual plants in the two F_2_ populations (2019 and 2020) were investigated to calculate the disease index. Using the ICIM method in QTL IciMapping Version 4.2, a linkage map was constructed based on genotyping and phenotyping data of the individuals. The results with a logarithm ratio of odds threshold score ≥ 3.0 were considered significant resistance QTLs.

### Histochemical staining

Tomato and tobacco plants were grown in the greenhouse, and leaf samples were collected at 7 dpi of TSWV. Visual detection of O_2_ and H_2_O_2_ was performed according to patterns and intensity of NBT and DAB staining, as reported previously [46]. TB staining was performed as reported previously [47]. The accumulation of H_2_O_2_ and O_2_ was quantified by image analysis of the stained sections using Image J software (V1.54f).

### RNA extraction and RT-qPCR

Transcript levels of target genes of different plants lines, including M82, R6, *SlDnaJ*/*Sldnaj*-silenced plants, 35S::*SlDnaJ/Sldnaj*-transformed tobacco plants, *SlDnaJ*-over line, and CRISPR/Cas9-edited lines, were analyzed by RT-qPCR with specific primers. Plants were cultured in the greenhouse in 2021 and total RNA was sampled from the young leaves at 0, 7, 14, 21, 28, and 35 dpi, with three samples collected per line. Beta-actin and elongation factor 1-α served as the standardized reference genes, and their primer sequences are listed in Table S8. Tomato total RNA was isolated with AG RNAex Pro RNA extraction reagent kit (Accurate Biotechnology Co., Ltd) and cDNA was synthesized with PrimeScript™ RT reagent kit (Takara Bio Inc.). The RT-qPCR was conducted on a Real-Time PCR System QuantStudio™ 5 (Applied Biosystems) using the SYBR Green Premix kit (Accurate Biotechnology Co., Ltd). Data were processed by the 2^−ΔΔCt^ method to determine differences in relative gene expression levels.

### Subcellular localization

The CDS of *SlDnaJ* without the stop codon was cloned and integrated into the pGreenII-35S-GFP plasmid. The recombinant plasmids were transformed into *Nicotiana benthamiana* leaves by *Agrobacterium* infiltration. After 3 days, the epidermis of tobacco leaves was examined under a fluorescence microscope to verify the subcellular localization of *SlDnaJ* protein.

### Relationship of the DnaJ family in tomato and polygenic analysis

The domain architecture of SlDnaJ protein was ‌evaluated using the SMART database. Phylogenetic analysis of SlDnaJ proteins was performed as previously described [48]. The phylogenetic tree of SlDnaJ proteins and 73 known DnaJ proteins was structured using the neighbor-joining linkage method in MEGA11 (V11.0.13). Three-dimensional structure models of SlDnaJ/Sldnaj proteins were analyzed using the AlphaFold v2 method in SWISS-MODEL (https://swissmodel.expasy.org/).

### VIGS-mediated silencing of candidate genes

The SGN VIGS Tool was used to design the VIGS vector constructs targeting *SlDnaJ/Sldnaj* sequences (Table S8). The *SlDnaJ/Sldnaj* sequences were designed in the R6 and M82 background. The sequence specificity was validated by genome-wide homology sequence comparison in Sol Genomics Network of BLAST. The TRV-based vectors pTRV1, empty TRV vector (pTRV:*00*), and pTRV:*SlDnaJ/Sldnaj* were individually injected with GV3101 *Agrobacterium*, as described previously [49]. Co-infiltration of plants and other operations were also conducted according to reported methods [12].

### Overexpression and gene knock-out

To perform overexpression analysis of *SlDnaJ*, the CDS was recombined into vector pBI121 and a recombinant plasmid 35S::*SlDnaJ*-CDS::GFP was constructed. The plasmid was then introduced into M82 plants to obtain overexpressed transgenic plants. To edit the *SlDnaJ* gene, two sgRNAs targeting the first exon of *SlDnaJ* were integrated into the CRIPSPR/Cas9 vector pCBC-DT1T2 to obtain the recombinant plasmid pHSE401 (Table S8). M82 (WT) explants were injected into the plasmids through the *Agrobacterium tumefaciens*-mediated transformation system [50]. The mutant plants were verified by genotyping the PCR products through sequencing.

### Phytohormone analysis

The phytohormone dynamics in reaction to TSWV invasion were analyzed in the *SlDnaJ*-overexpression line, CRISPR/Cas9-edited lines, and WT plants inoculated with TSWV. Hormone levels of fresh tomato leaves sampled at 0, 7, 14, 28, and 35 dpi were measured using liquid chromatography–mass spectrometry with the AB SCIEX QTRAP^®^ 5500 system, as described previously [51]. The phytohormone contents were determined based on a previously described method [12].

### Activity of antioxidant enzymes

The POD, SOD, and CAT enzyme activities were detected by the BC5165, BC0095, and BC0205 assay kits (Solarbio), respectively, at 0, 7, 14, 28, and 35 dpi in the WT, *SlDnaJ*-overexpression line, and CRISPR/Cas9-edited *Sldnaj*-cr line in accordance with the manufacturer’s instructions.

### Statistical analysis

To evaluate the data statistically, Fishers LSD test was performed in SPSS 26.0. The means ± standard deviation were graphed using OriginPro version 2022 from three independent biological replicates. The statistical significance of the data between the control and treatment groups was evaluated by Student’s *t*-test.

## Supplementary Material

Web_Material_uhaf019

## Data Availability

All the relevant data can be found within this manuscript and its supporting information files.
