# Supplementary material for: Association of the tomato co-chaperone gene Sldnaj harboring a promoter deletion with susceptibility to Tomato spotted wilt virus (TSWV)
Source: Hortic Res. 2025 Jan 15;12(4):uhaf019. doi: 10.1093/hr/uhaf019 (PMC11908825; doi:10.1093/hr/uhaf019)
Supplement: Web_Material_uhaf019 [file web_material_uhaf019.zip › Supplementary FigureS1-S15 revised Editor clean version 20241207.pdf]

Association of the tomato co-chaperone gene *Sldnaj* harboring a promoter deletion with susceptibility to Tomato spotted wilt virus (TSWV)

Supplementary Data

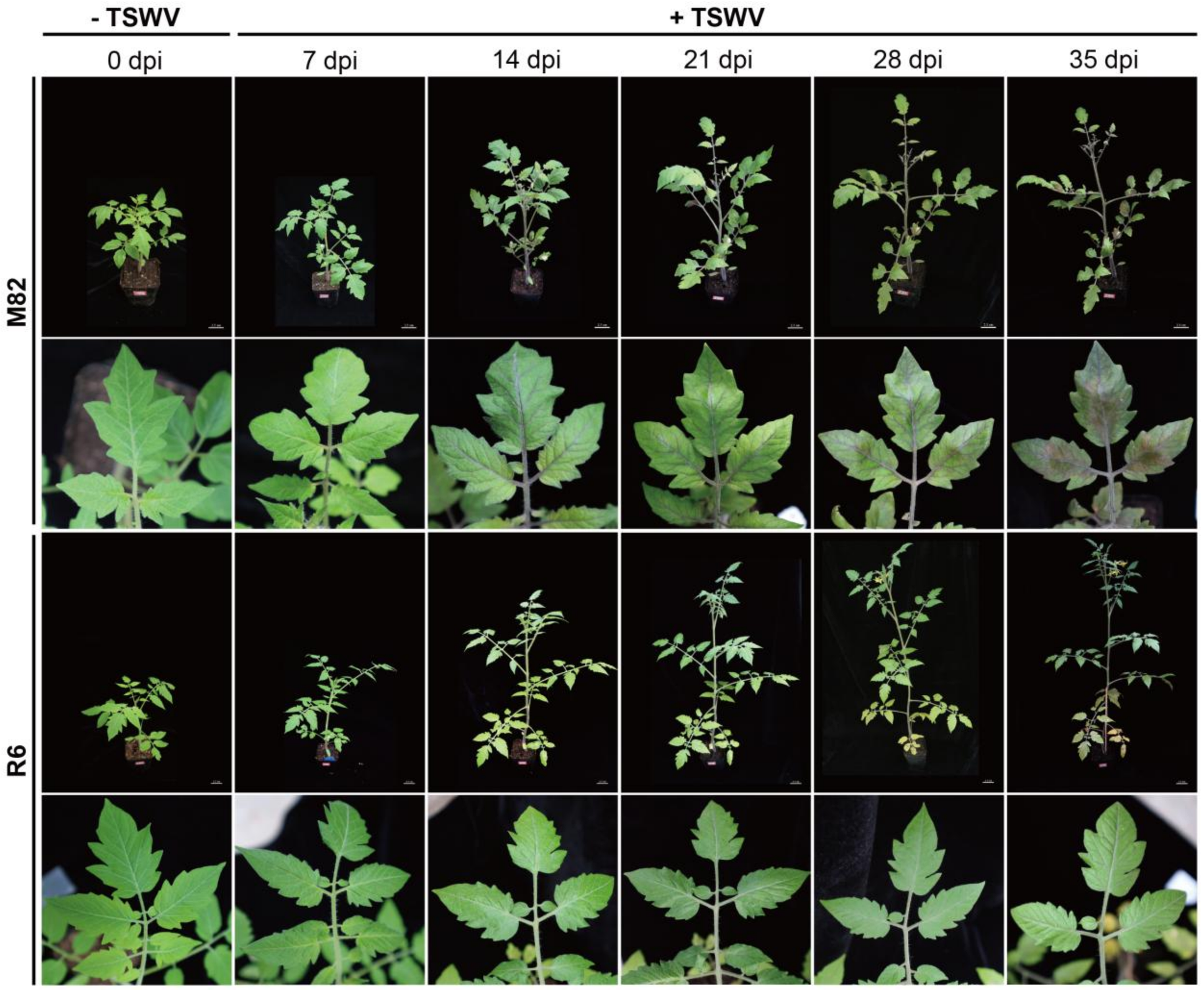

Figure S1. The performance of M82 and R6 plants after infection with TSWV at 0, 7, 14, 21, 28, and 35 days post-infection (dpi). Scale bar, 25 mm.

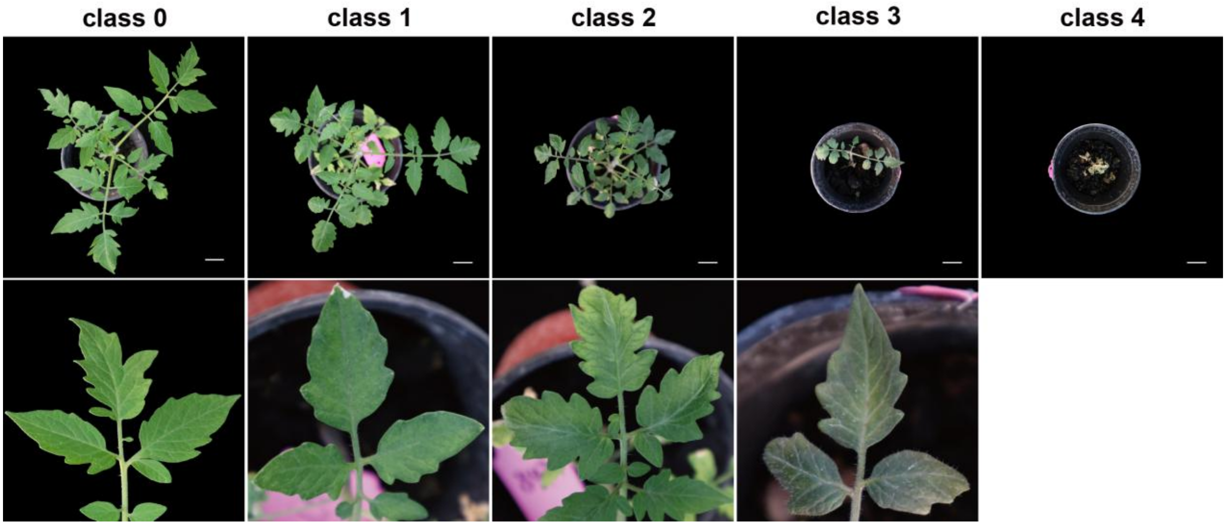

Figure S2. The disease severity scored from class 0 to 4 based on the F_2 population plants (R6×M82 tomato lines). The white line segment indicates the scale bar = 3 cm.

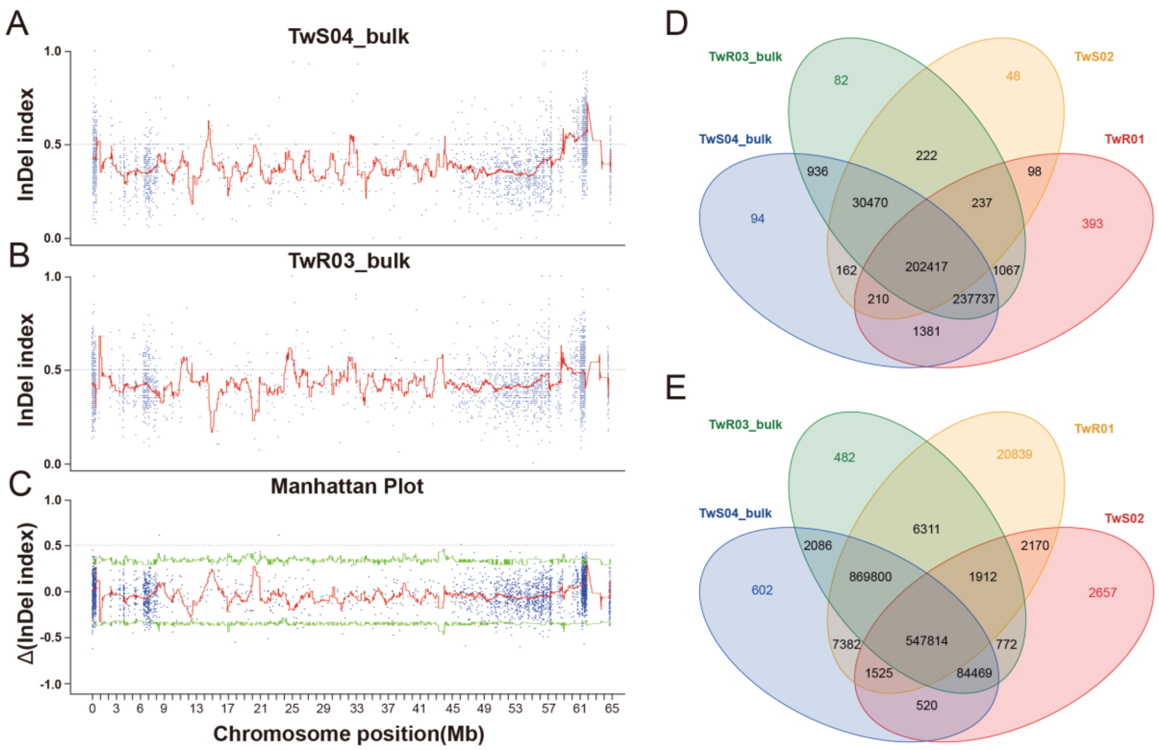

Figure S3. Distribution of progeny Δ(InDel index) and differences in the InDel and SNP Wayne diagram. Distribution of the InDel index of TwR01 **(A)** TwS02 **(B)** on chromosome 10. (**C**) Distribution of the Δ(InDel index) in the two progeny lines (TwR03_bulk and TwS04_bulk) on chromosome 10. (**D**) InDel and (**E**) SNP differences in the parents (TwR01 and TwS02) and progeny (TwR03_bulk and TwS04_bulk) Wayne diagram. TwR01, R6 plants. TwS02, M82 plants. TwR03_bulk, mix of 30 highly resistant F_2 individuals. TwS04_bulk, mix of 30 highly susceptible F_2 individuals.

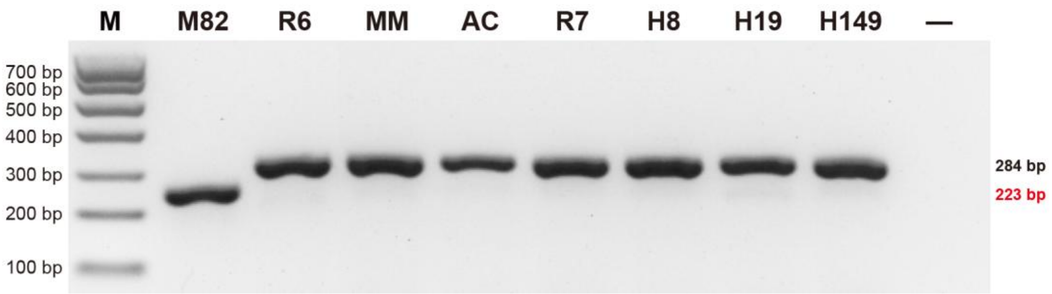

17 **Figure S4.** PCR results for different tomato plants using the marker InDc10_221. M, Trans
18 DNA Marker I. MM, Moneymaker; AC, Alisa Craig.

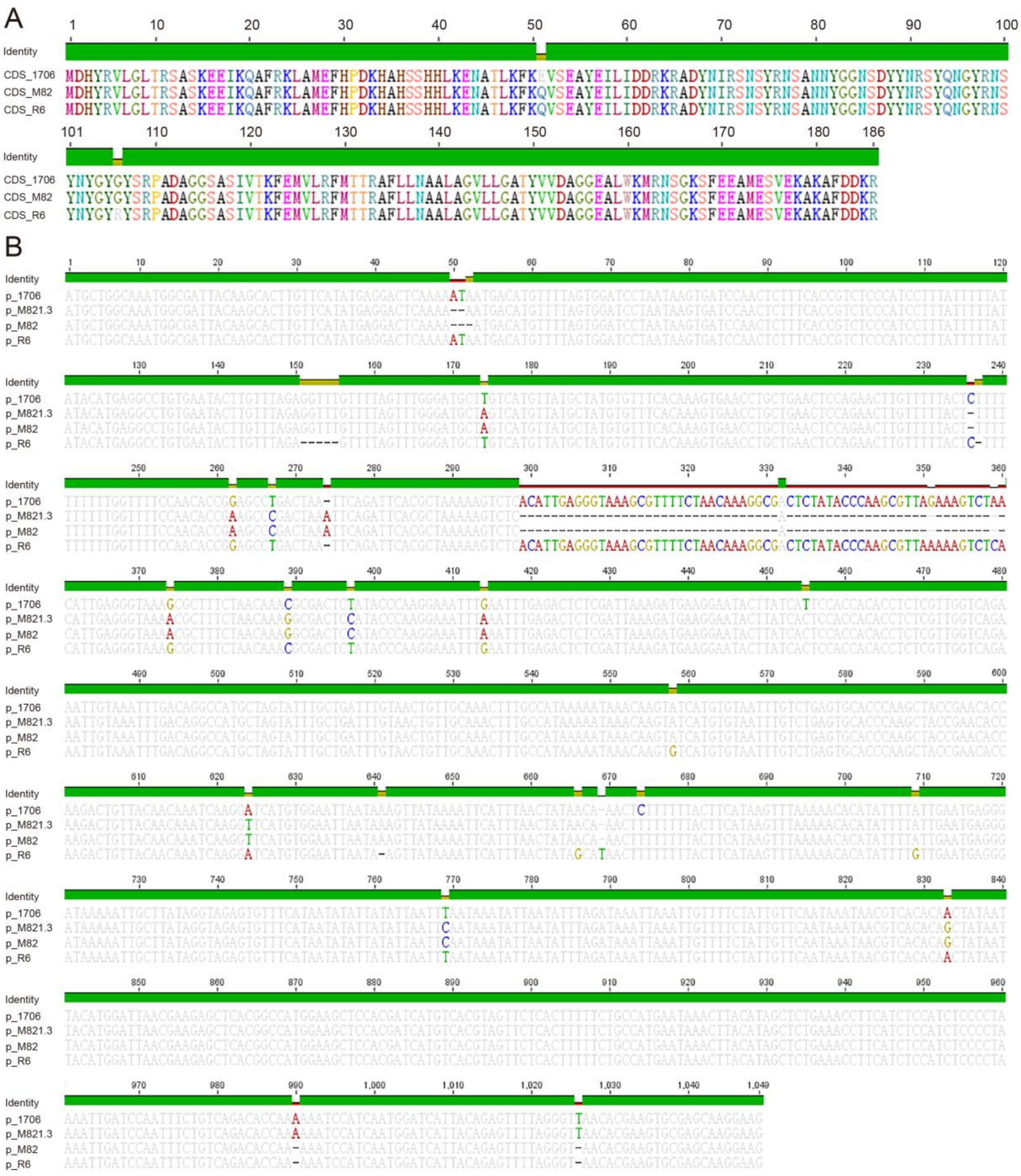

Figure S5. Blast of the coding sequence (CDS) and promoter sequence of *SlDnaJ* between M82 and R6 plants. (**A**) Blast of the CDS of *SlDnaJ* between M82 and R6 plants. (**B**) Blast of the promoter sequence of *SlDnaJ* between M82 and R6 plants. Sequence alignment reference genome version: SL4.0 (Heinz 1706), M821.3 (M82).

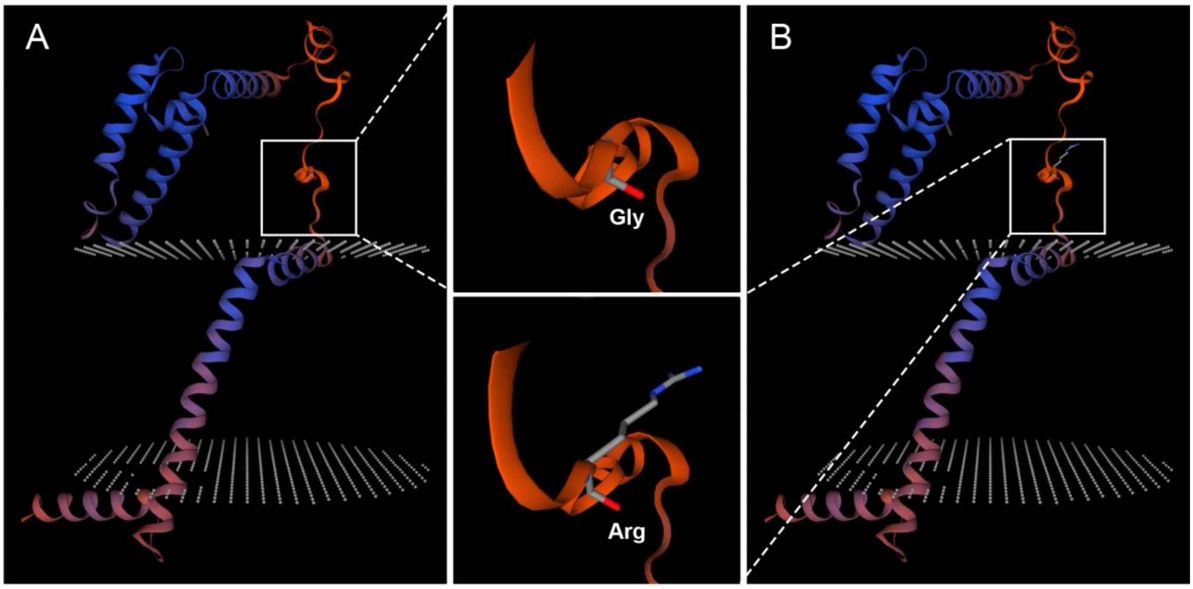

Figure S6. Three-dimensional structure models of SlDnaJ/Sldnaj proteins. (**A**) Three-dimensional structure models of Sldnaj protein using SWISS-MODEL. The red-gray stick represents glycine (Gly, G). Global model quality estimate (GMQE): 0.70; sequence identity: 87.57%; coverage: 0.99. (**B**) Three-dimensional structure models of SlDnaJ protein using SWISS-MODEL. The red-gray-blue stick represents arginine (Arg, R). Global model quality estimate (GMQE): 0.70; sequence identity: 87.03%; coverage: 0.99.

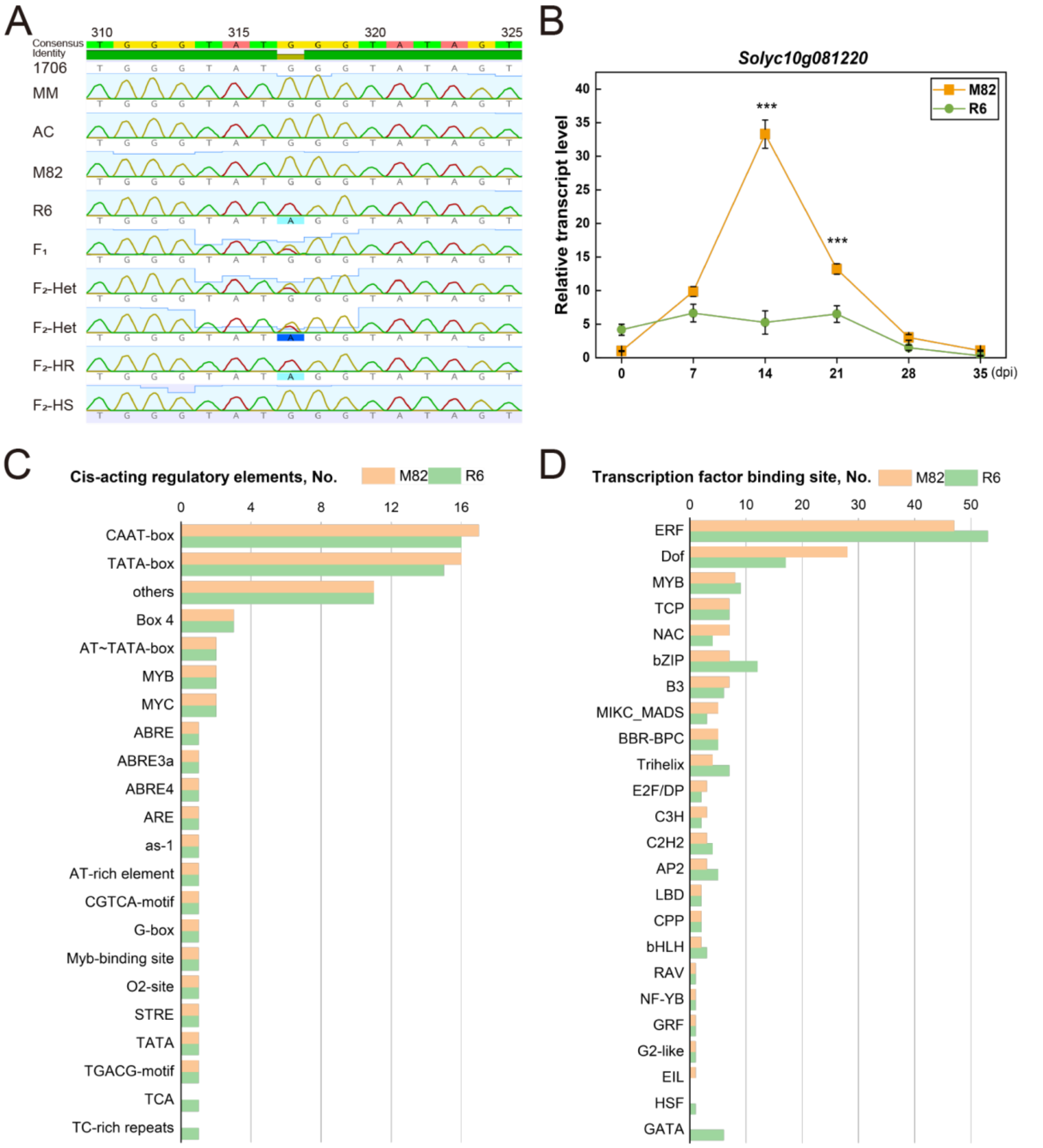

Figure S7. The mutant bases and without deletion promoter sequences in *SlDnaJ* are highly conserved, and the expression of *SlDnaJ* is significantly different in susceptible and resistant plants. **(A)** The mutated base in the coding region of *SlDnaJ* was sequenced in the M82, R6, Heinz 1706, Moneymaker (MM), Alisa Craig (AC), F_2-heterozygous (F_2-Het), F_2-homozygous resistant (F_2-HR), and F_2-homozygous susceptible (F_2-HS) plants. **(B)** RT-qPCR analysis of *SlDnaJ* expression in M82 and R6 plants at 0, 7, 14, 21, 28, and 35 days post-inoculation (dpi). The error bars represent the standard deviations of three biological replicates. Three asterisks mean significant differences at $P < 0.001$. (**C**) Distribution of cis regulatory elements related to the defense response in the *SlDnaJ* promoter region of M82 and R6 plants, as identified using the PlantCARE database. **(D)** The transcription factor-binding sites in the *SlDnaJ* promoter of M82 and R6 predicted by PlantRegMap.

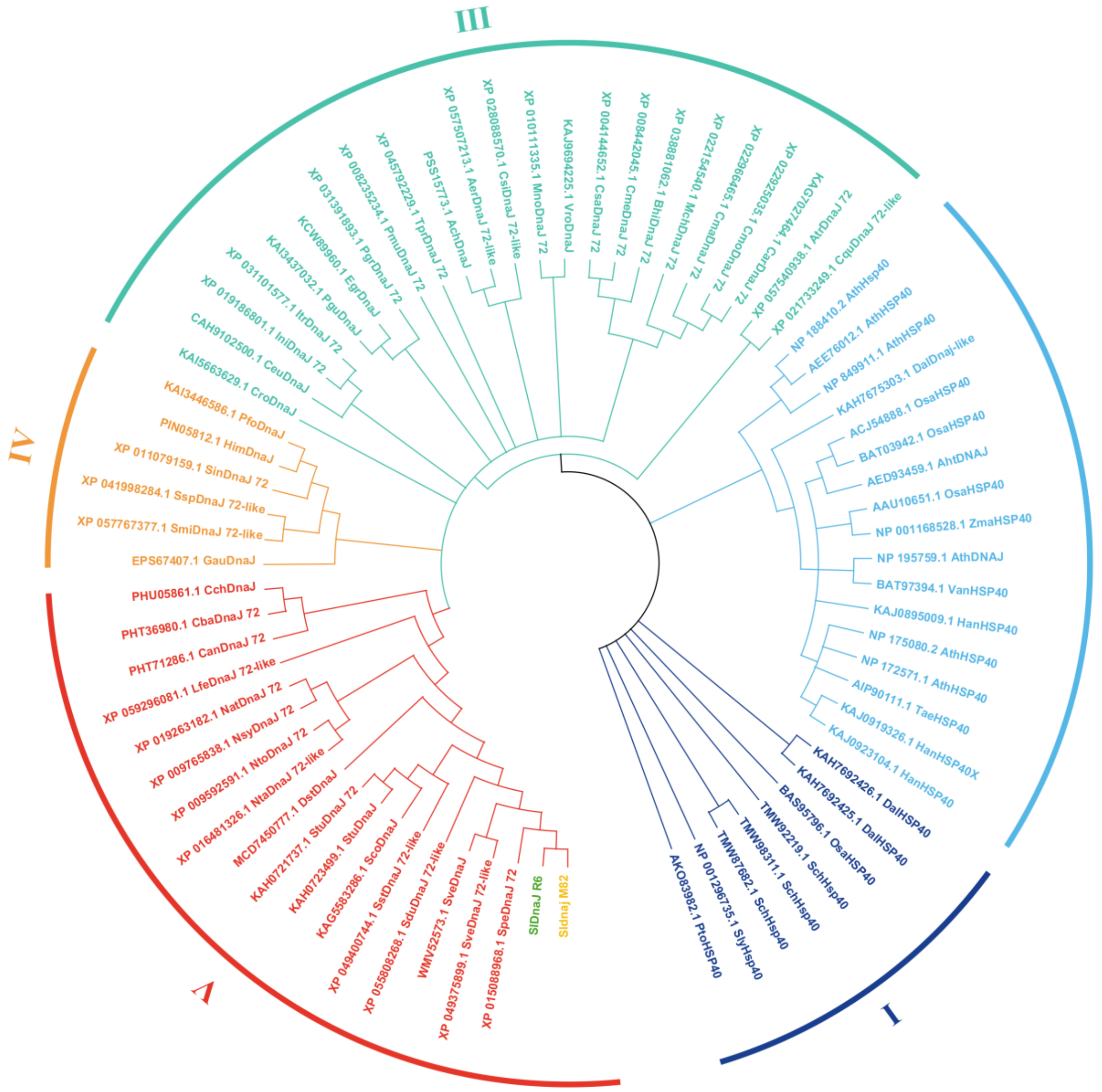

Figure S8. Phylogenetic relationships among SlDnaJ proteins inferred using the neighbor-joining method. Three-letter abbreviations refer to species names: Ach, *Actinidia chinensis*; Aer, *Actinidia eriantha*; Ath, *Arabidopsis thaliana*; Atr, *Amaranthus tricolor*; Bhi, Benincasa hispida; Can, Capsicum annuum; Car, *Cucurbita argyrosperma*; Cba, *Capsicum baccatum*; Cch, *Capsicum chinense*; Ceu, *Cuscuta europaea*; Cma, *Cucurbita maxima*; Cme, *Cucumis melo*; Cmo, *Cucurbita moschata*; Cqu, *Chenopodium quinoa*; Cro, *Catharanthus roseus*; Csa, *Cucumis sativus*; Csi, *Camellia sinensis*; Dal, *Dioscorea alata*; Dst, *Datura stramonium*; Egr, *Eucalyptus grandis*; Gau, *Genlisea aurea*; Han, *Helianthus annuus*; Him, *Handroanthus impetiginosus*; Ini, *Ipomoea nil*; Itr, *Ipomoea triloba*; Lfe, *Lycium ferocissimum*; Mch, Momordica charantia; Mno, Morus notabilis; Nat, *Nicotiana attenuata*; Nsy, Nicotiana sylvestris; Nta, Nicotiana tabacum; Nto, *Nicotiana tomentosiformis*; Osa, *Oryza sativa Japonica*; Pfo, *Paulownia fortunei*; Pgr, *Punica granatum*; Pgu, *Psidium guajava*; Pmu, *Prunus mume*; Pto, *Populus tomentosa*; Sch, Solanum chilense; Sco, *Solanum commersonii*; Sdu,

Solanum dulcamara; Sin, *Sesamum indicum*; Sly, *Solanum lycopersicum*; Smi, *Salvia miltiorrhiza*; Spe, *Solanum pennellii*; Ssp, *Salvia splendens*; Sst, *Solanum stenotomum*; Stu, *Solanum tuberosum*; Sve, *Solanum verrucosum*; Tae, Triticum aestivum; *Tpr, Trifolium pratense*; Van, *Vigna angularis*; Vro, *Vitis rotundifolia*; Zma, *Zea mays*.

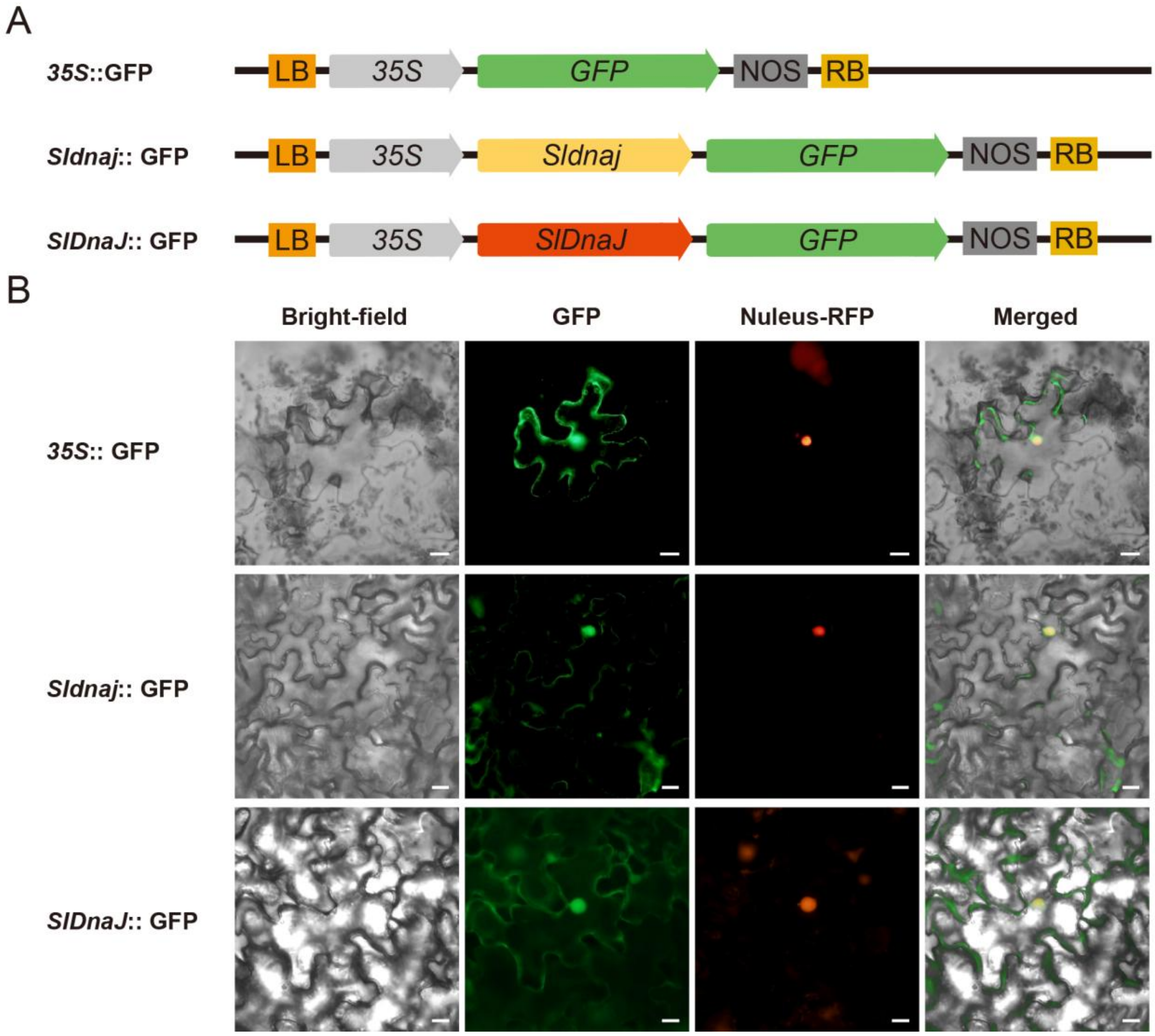

Figure S9. Subcellular localization of *Sldnaj* and *SlDnaJ*. **(A)** Diagram of the *Sldnaj* and *SlDnaJ* subcellular localization vector's structure. **(B)** Subcellular localization of *Sldnaj* and *SlDnaJ*. 35S:*Sldnaj*-GFP and 35S:*SlDnaJ*-GFP represent *Sldnaj* and *SlDnaJ* fused to a green fluorescence protein (GFP), respectively. The GFP driven by the 35S promoter (35S:GFP) served as the negative control. The crocus points denote nuclei. Scale bars, 20 µm.

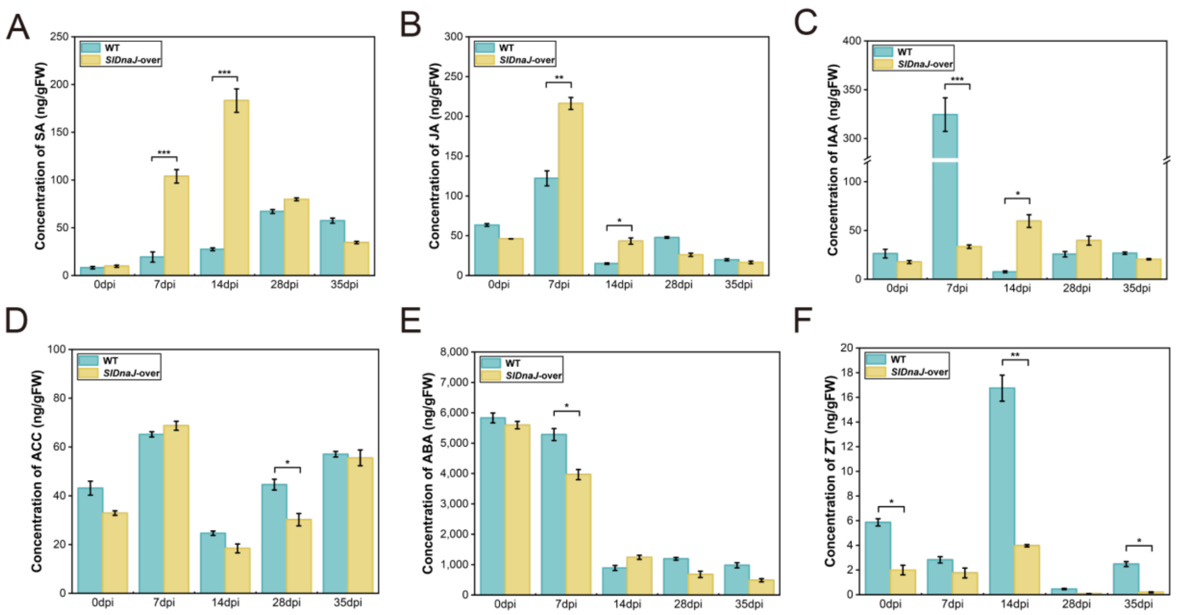

Figure S10. Phytohormone production in the over-expression *SlDnaJ* line inoculated with TSWV. (**A**–**F**) Levels of SA, JA, IAA, ACC, ABA, and ZT extracted from the leaf tissue of plants [wild-type (WT) and the *SlDnaJ*-overexpression line] at 0, 7, 14, 28, and 35 days post-injection (dpi) of TSWV. The contents of SA, JA, IAA, ACC, ABA, and ZT were recorded as nanograms per gram of fresh weight (ng/gFW). The error bars represent the standard deviations of three biological replicates. One asterisk, two asterisks, and three asterisks indicate significant differences at $P < 0.05$, $P < 0.01$, and $P < 0.001$, respectively.

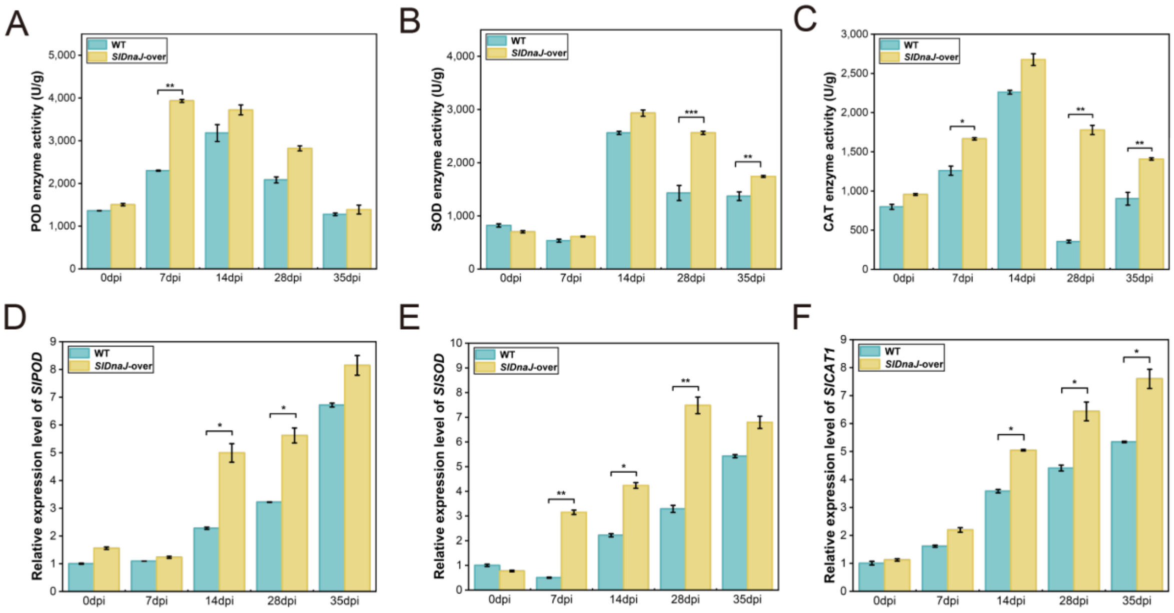

Figure S11. Activity of antioxidant enzymes and the related gene expression levels in the over-expression *SlDnaJ* line inoculated with TSWV. (**A**–**C**) POD, SOD, and CAT enzyme activities at 0, 7, 14, 28, and 35 dpi in the wild type (WT) and *SlDnaJ*-overexpression line. (**D**–**F**) Transcript levels of *SlPOD*, *SlSOD*, and *SlCAT* in the *SlDnaJ*-overexpression line at 0, 7, 14, 28, and 35 dpi analyzed using qRT-PCR. The transcript levels were normalized to the level of β-actin. The error bars represent the standard deviations of three biological replicates. One asterisk, two asterisks, and three asterisks indicate significant differences at $P < 0.05$, $P < 0.01$, and $P < 0.001$, respectively.

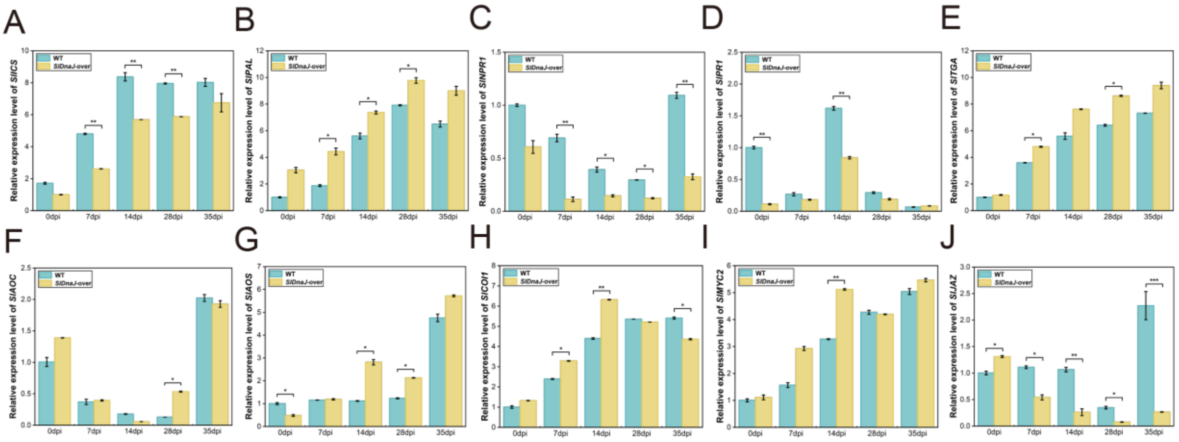

Figure S12. The transcript levels of key SA/JA-signaling pathway genes in the *SlDnaJ*-overexpression line inoculated with TSWV. (**A–E**) RT-qPCR analysis to determine the transcript levels of SA-signaling pathway genes in the *SlDnaJ*-overexpression line at 0, 7, 14, 28, and 35 dpi. (**F–J**) RT-qPCR analysis to determine the transcript levels of JA-signaling pathway genes in the *SlDnaJ*-overexpression line at 0, 7, 14, 28, and 35 dpi. The expression levels were normalized to the level of β-actin. The error bars represent the standard deviations of three biological replicates. One asterisk, two asterisks, and three asterisks indicate significant differences at $P < 0.05$, $P < 0.01$, and $P < 0.001$, respectively.

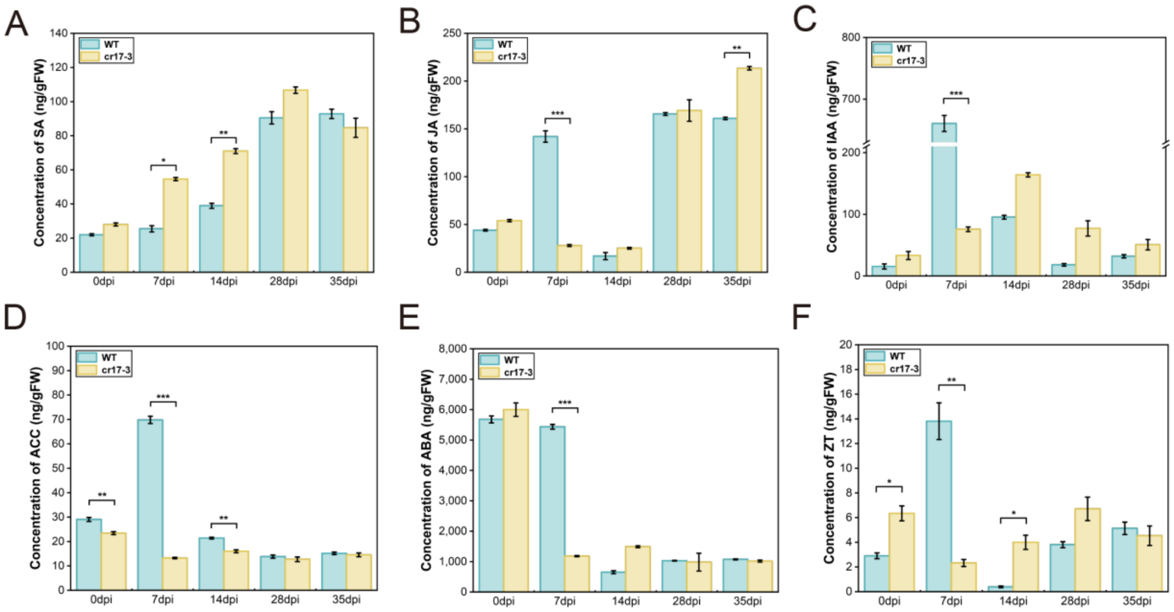

Figure S13. Phytohormone production in the CRISPR/Cas9-edited *Sldnaj*-cr17-3 line inoculated with TSWV. (**A**–**F**) Levels of SA, JA, IAA, ACC, ABA, and ZT extracted from the leaf tissue of plants [wild type (WT) and the Sldnaj-cr17-3 line] at 0, 7, 14, 28, and 35 days post-inoculation (dpi) of TSWV. The contents of SA, JA, IAA, ACC, ABA, and ZT were recorded as nanograms per gram of fresh weight (ng/gFW). The error bars represent the standard deviations of three biological replicates. One asterisk, two asterisks, and three asterisks indicate significant differences at $P < 0.05$, $P < 0.01$, and $P < 0.001$, respectively.

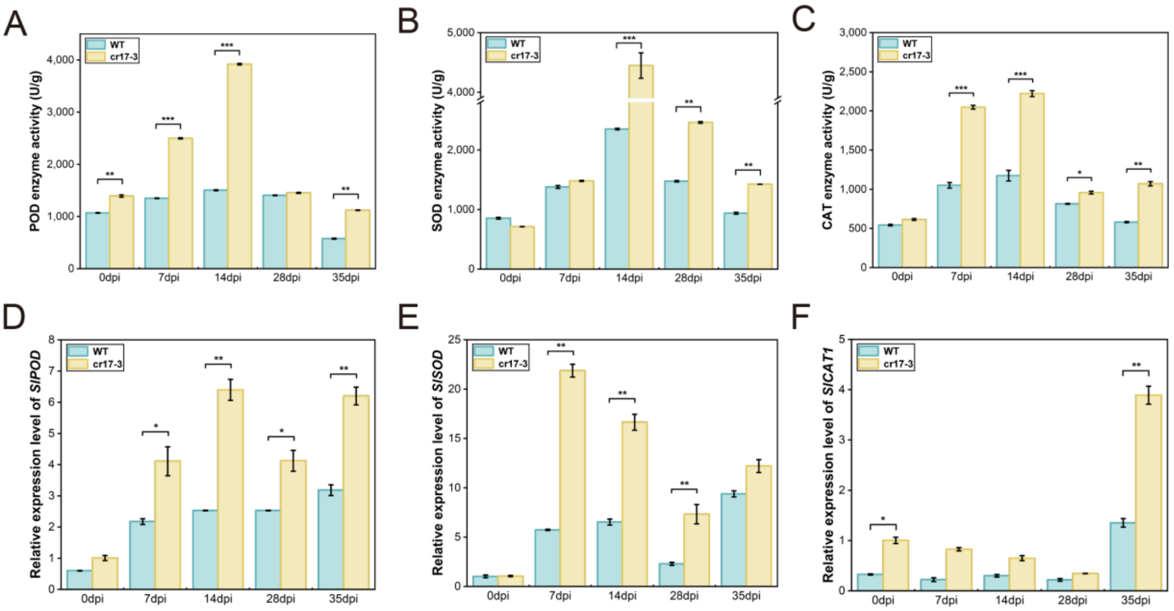

Figure S14. Activity of antioxidant enzymes and transcript levels of related genes in the CRISPR/Cas9-edited *Sldnaj*-cr17-3 line inoculated with TSWV. (**A–C**) The POD, SOD, and CAT enzyme activities at 0, 7, 14, 28, and 35 dpi in the wild type (WT) and the *Sldnaj*-cr17-3 line inoculated with TSWV. (**D–F**) Transcript levels of *SlPOD*, *SlSOD*, and *SlCAT* in the *Sldnaj*-cr17-3 line at 0, 7, 14, 28, and 35 dpi analyzed using qRT-PCR. The expression levels were normalized to the level of β-actin. The error bars represent the standard deviations of three biological replicates. One asterisk, two asterisks, and three asterisks indicate significant differences at $P < 0.05$, $P < 0.01$, and $P < 0.001$, respectively.

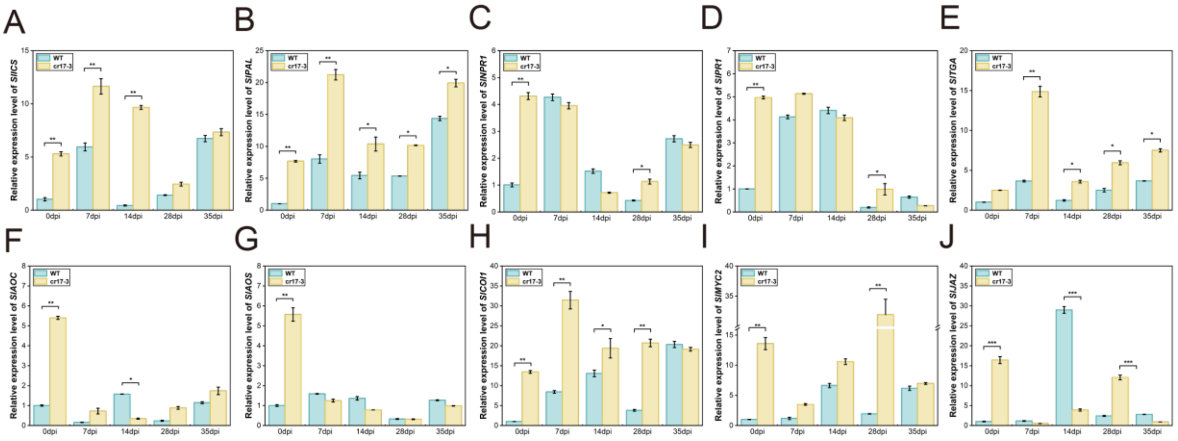

Figure S15. The transcript levels of key SA/JA-signaling pathway genes in the CRISPR/Cas9-edited *Sldnaj*-cr17-3 line inoculated with TSWV. (**A–E**) RT-qPCR analysis to determine the transcript levels of SA-signaling pathway genes in the *Sldnaj*-cr17-3 line at 0, 7, 14, 28, and 35 dpi. (**F–J**) RT-qPCR analysis to determine the transcript levels of JA-signaling pathway genes in the *Sldnaj*-cr17-3 line at 0, 7, 14, 28, and 35 dpi. The expression levels were normalized using β-actin as an internal reference gene. The error bars represent the standard deviations of three biological replicates. One asterisk, two asterisks, and three asterisks mean significant differences at $P < 0.05$, $P < 0.01$, and $P < 0.001$, respectively.

The TSWV susceptible gene *Sldnaj* CDS sequence in M82 plant

1 ATGGATCATT ACAGAGTTTT AGGGTTAACA CGAAGTGCGA GCAAGGAAGA
51 AATCAAGCAA GCGTTTCGGA AATTGGCAAT GGAATTTCAT CCCGACAAGC
101 ACGCACATTC TTCGCATCAC TTGAAGGAAA ACGCTACGCT TAAATTCAAG
151 CAAGTTTCCG AAGCTTACGA GATTCTGATC GACGATCGCA AACGCGCTGA
201 TTACAATATC CGGTCTAATA GTTACCGGAA TTCAGCGAAT AATTATGGCG
251 GTAATAGTGA CTATTATAAT CGTAGTTATC AGAACGGGTA TAGAAATAGC
301 TACAATTATG GGTATGGGTA TAGTCGGCCT GCTGATGCCG GCGGTTCTGC
351 AAGTATTGTT ACCAAATTCG AGATGGTTCT GCGGTTCATG ACGACAAGGG
401 CGTTTCTCCT CAATGCTGCA CTTGCCGGTG TATTGTTAGG TGCAACATAT
451 GTAGTTGATG CAGGTGGGGA GGCACTATGG AAGATGCGAA ATTCTGGAAA
501 ATCGTTTGAA GAAGCAATGG AATCTGTAGA AAAAGCTAAA GCATTTGATG
551 ACAAAAGA

The TSWV resistance gene *SlDnaJ* (Solyc10g081220) CDS sequence in R6 plant

1 ATGGATCATT ACAGAGTTTT AGGGTTAACA CGAAGTGCGA GCAAGGAAGA
51 AATCAAGCAA GCGTTTCGGA AATTGGCAAT GGAATTTCAT CCCGACAAGC
101 ACGCACATTC TTCGCATCAC TTGAAGGAAA ACGCTACGCT TAAATTCAAG
151 CAAGTTTCCG AAGCTTACGA GATTCTGATC GACGATCGCA AACGCGCTGA
201 TTACAATATC CGGTCTAATA GTTACCGGAA TTCAGCGAAT AATTATGGCG
251 GTAATAGTGA CTATTATAAT CGTAGTTATC AGAACGGGTA TAGAAATAGC
301 TACAATTATG GGTATAGGTA TAGTCGGCCT GCTGATGCCG GCGGTTCTGC
351 AAGTATTGTT ACCAAATTCG AGATGGTTCT GCGGTTCATG ACGACAAGGG
401 CGTTTCTCCT CAATGCTGCA CTTGCCGGTG TATTGTTAGG TGCAACATAT
451 GTAGTTGATG CAGGTGGGGA GGCACTATGG AAGATGCGAA ATTCTGGAAA
501 ATCGTTTGAA GAAGCAATGG AATCTGTAGA AAAAGCTAAA GCATTTGATG
551 ACAAAAGA
