# Supplementary figures and images for: Association of the tomato co-chaperone gene Sldnaj harboring a promoter deletion with susceptibility to Tomato spotted wilt virus (TSWV)
Source: Hortic Res. 2025 Jan 15;12(4):uhaf019. doi: 10.1093/hr/uhaf019 (PMC11908825; doi:10.1093/hr/uhaf019)

- TSWV

+ TSWV

0 dpi

7 dpi

14 dpi

21 dpi

28 dpi

35 dpi

M82

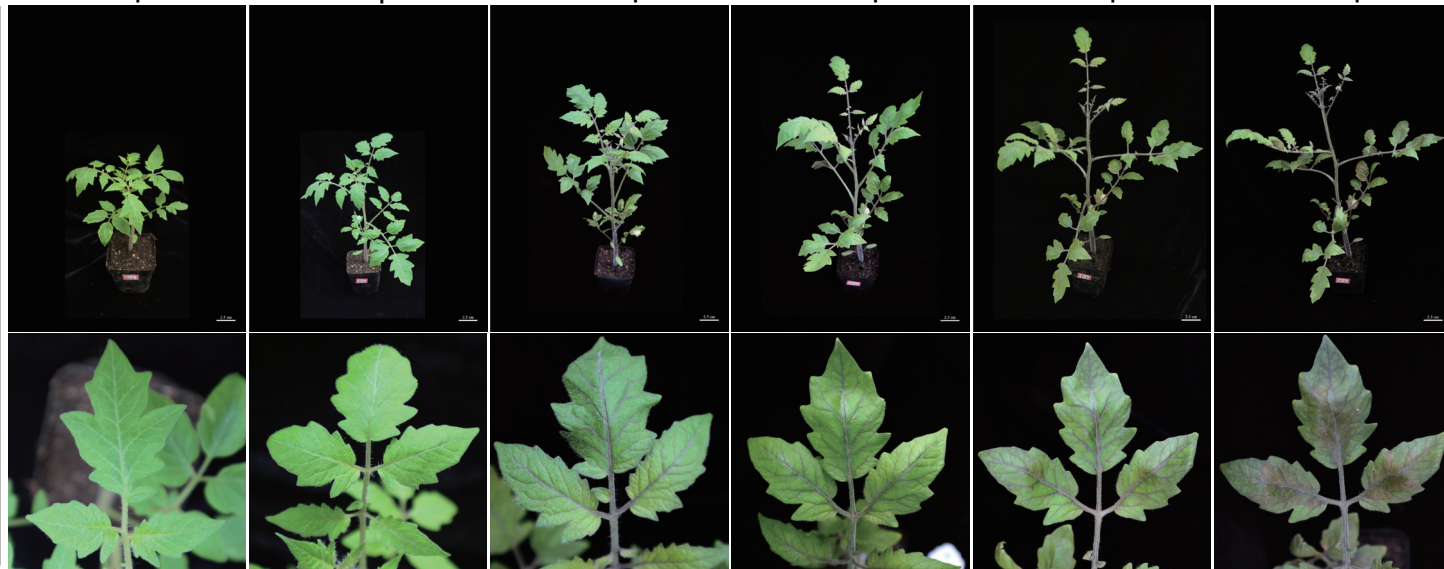

R6

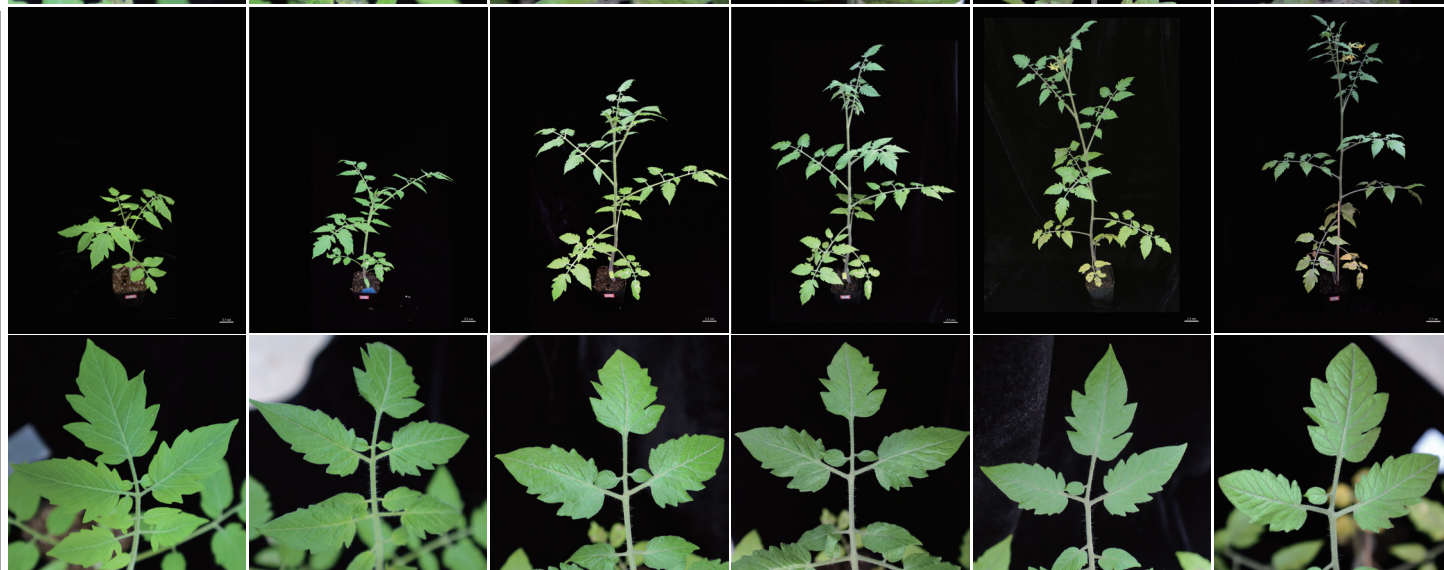

Supplement: Web_Material_uhaf019 [file web_material_uhaf019.zip › Fig. S1.pdf]

**class 0**

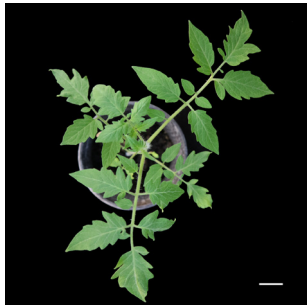

**class 1**

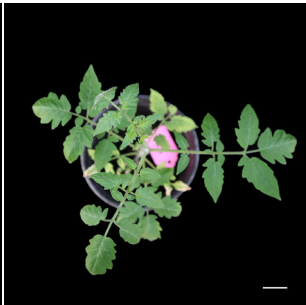

**class 2**

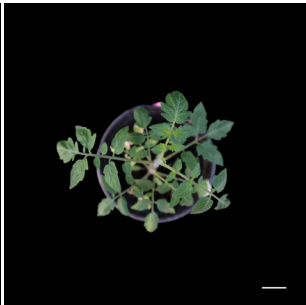

**class 3**

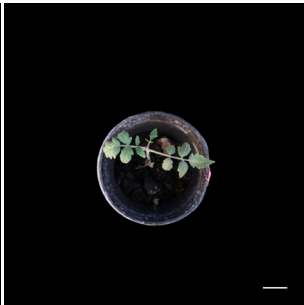

**class 4**

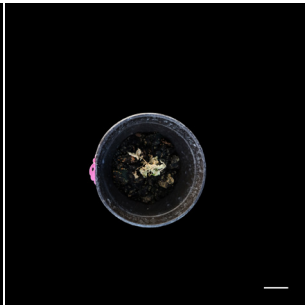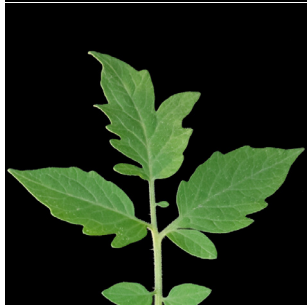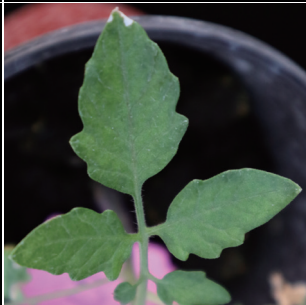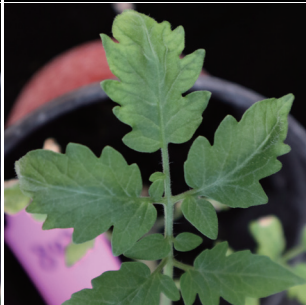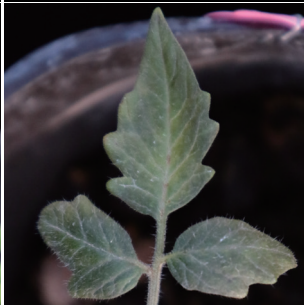

Supplement: Web_Material_uhaf019 [file web_material_uhaf019.zip › Fig. S2.pdf]

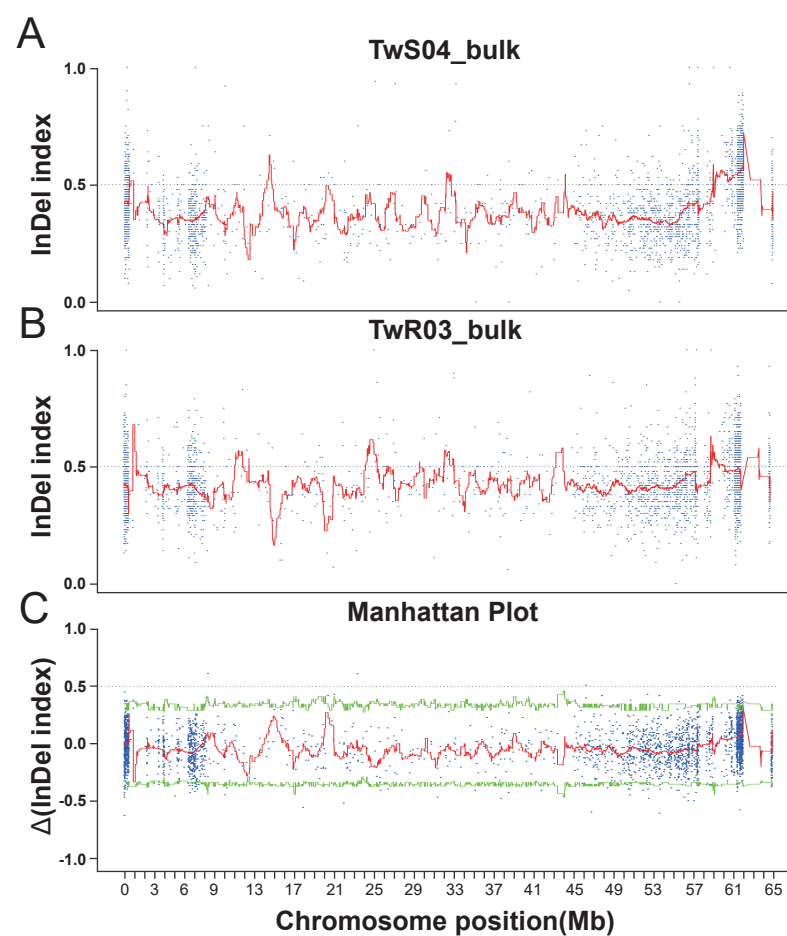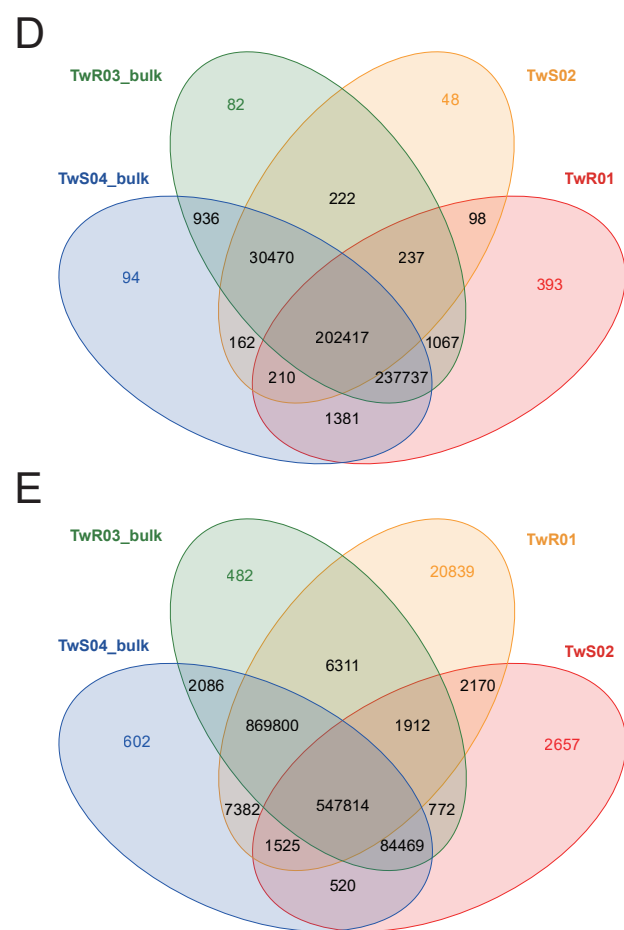

Supplement: Web_Material_uhaf019 [file web_material_uhaf019.zip › Fig. S3.pdf]

**M      M82      R6      MM      AC      R7      H8      H19      H149      —**

700 bp  
600 bp  
500 bp  
400 bp  
300 bp  
200 bp  
100 bp

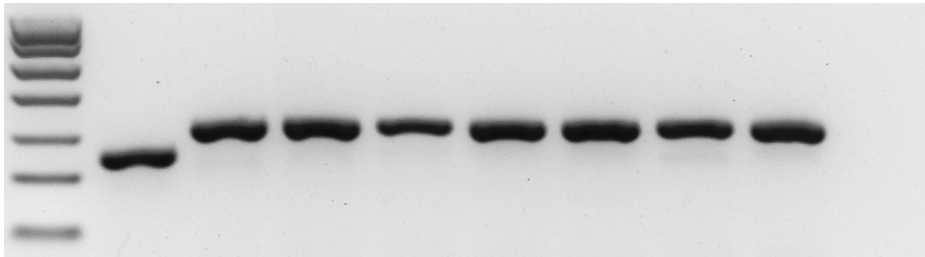

284 bp

223 bp

Supplement: Web_Material_uhaf019 [file web_material_uhaf019.zip › Fig. S4.pdf]

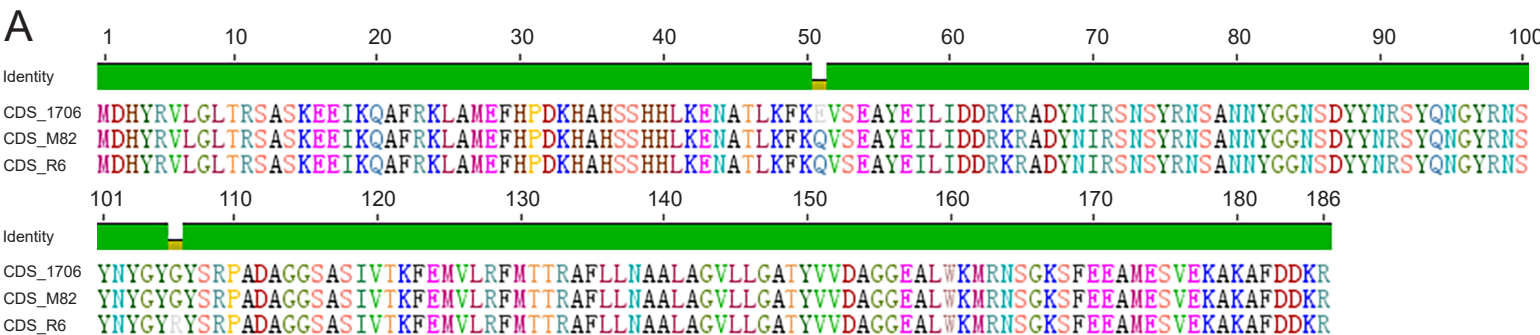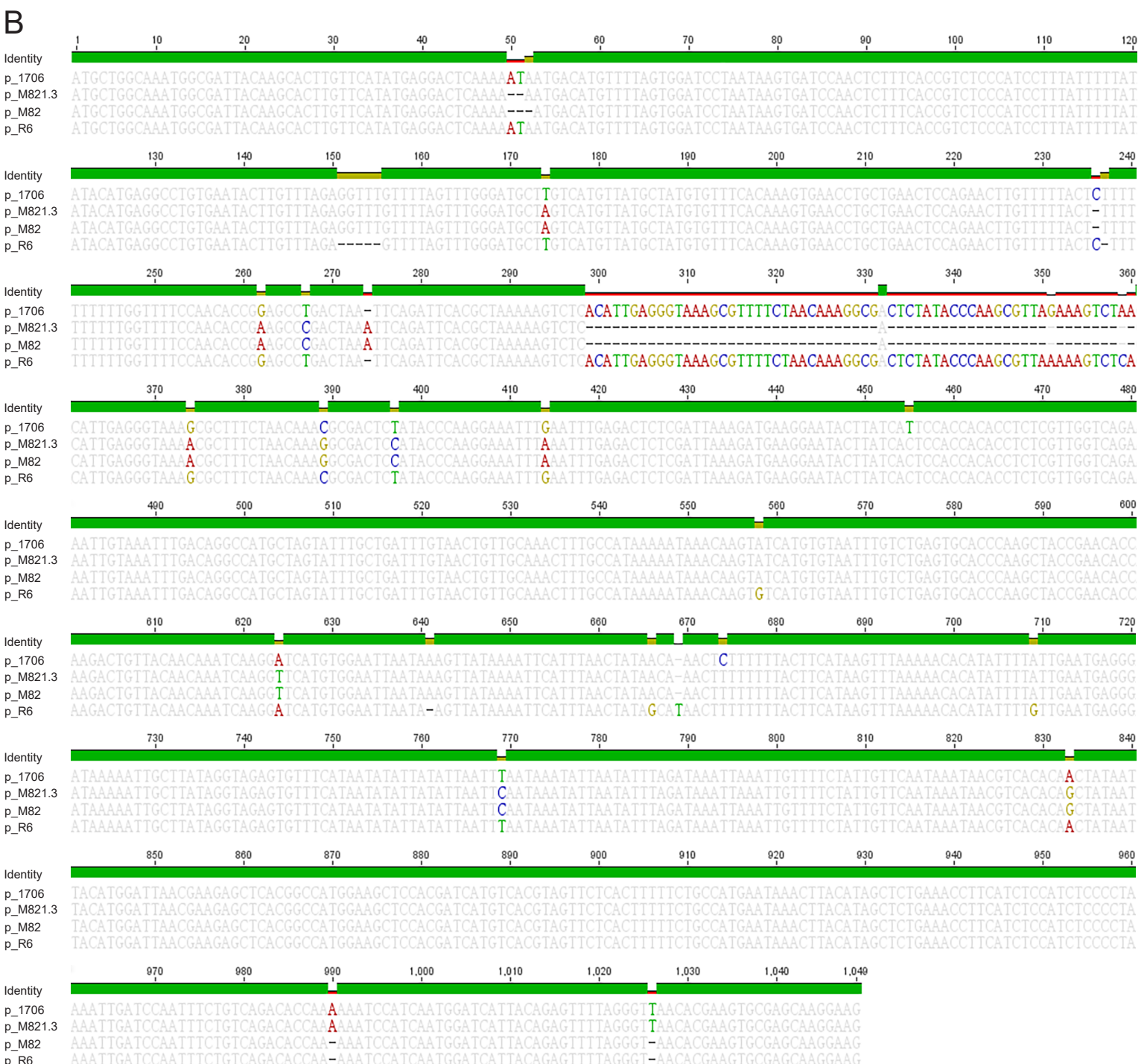

Supplement: Web_Material_uhaf019 [file web_material_uhaf019.zip › Fig. S5.pdf]

A

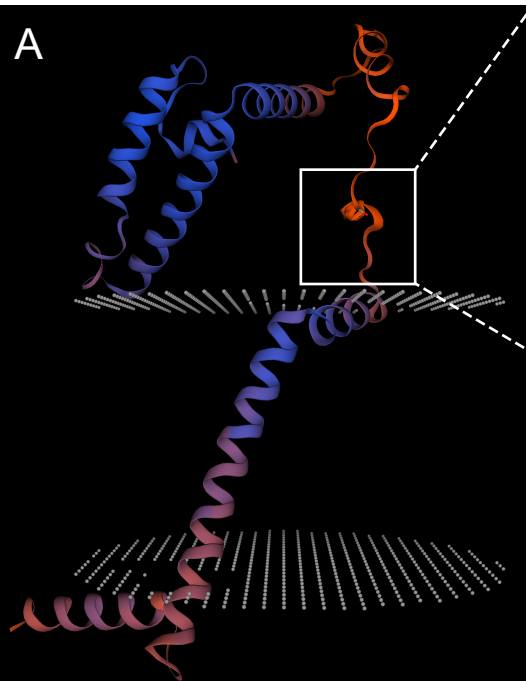

Gly

Arg

B

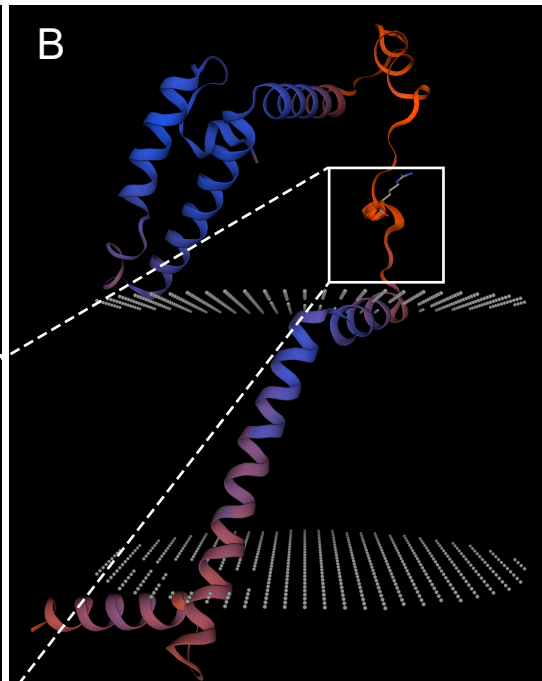

Supplement: Web_Material_uhaf019 [file web_material_uhaf019.zip › Fig. S6 revised.pdf]

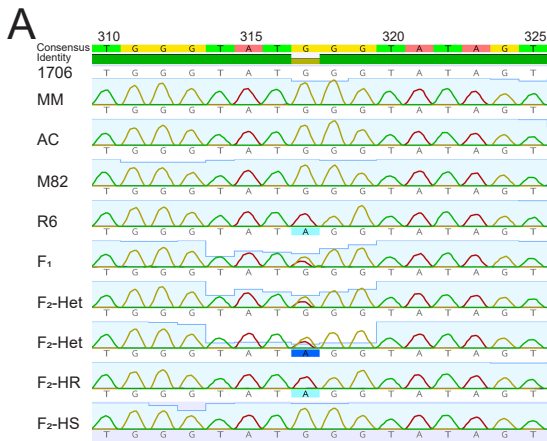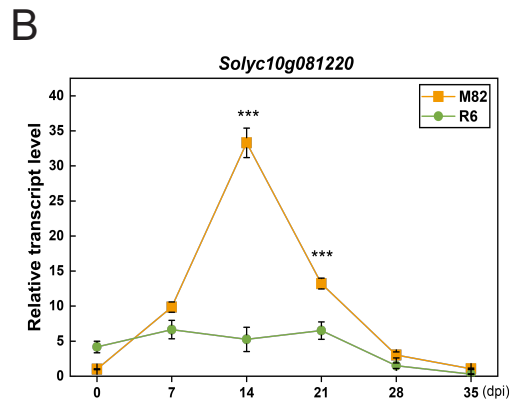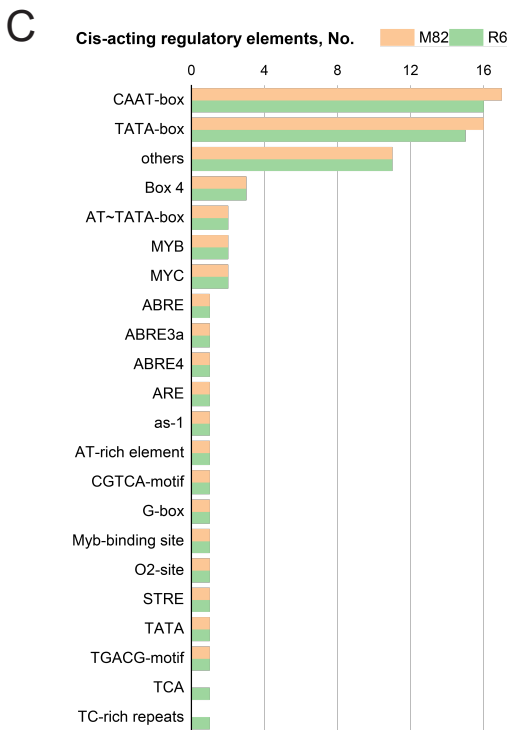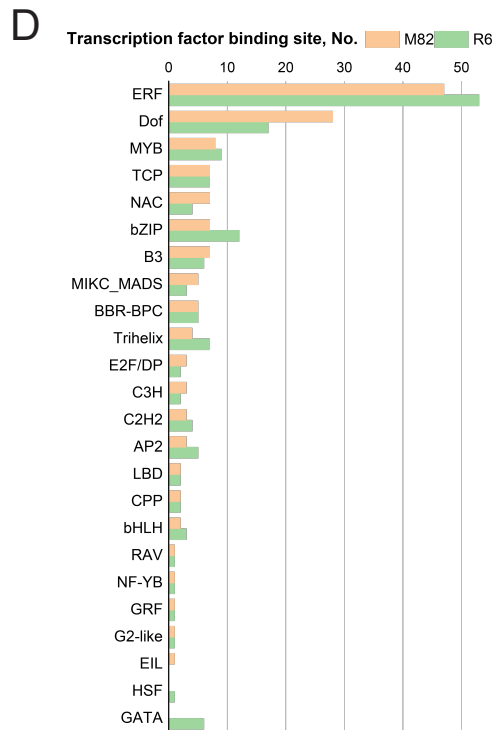

Supplement: Web_Material_uhaf019 [file web_material_uhaf019.zip › Fig. S7+.pdf]

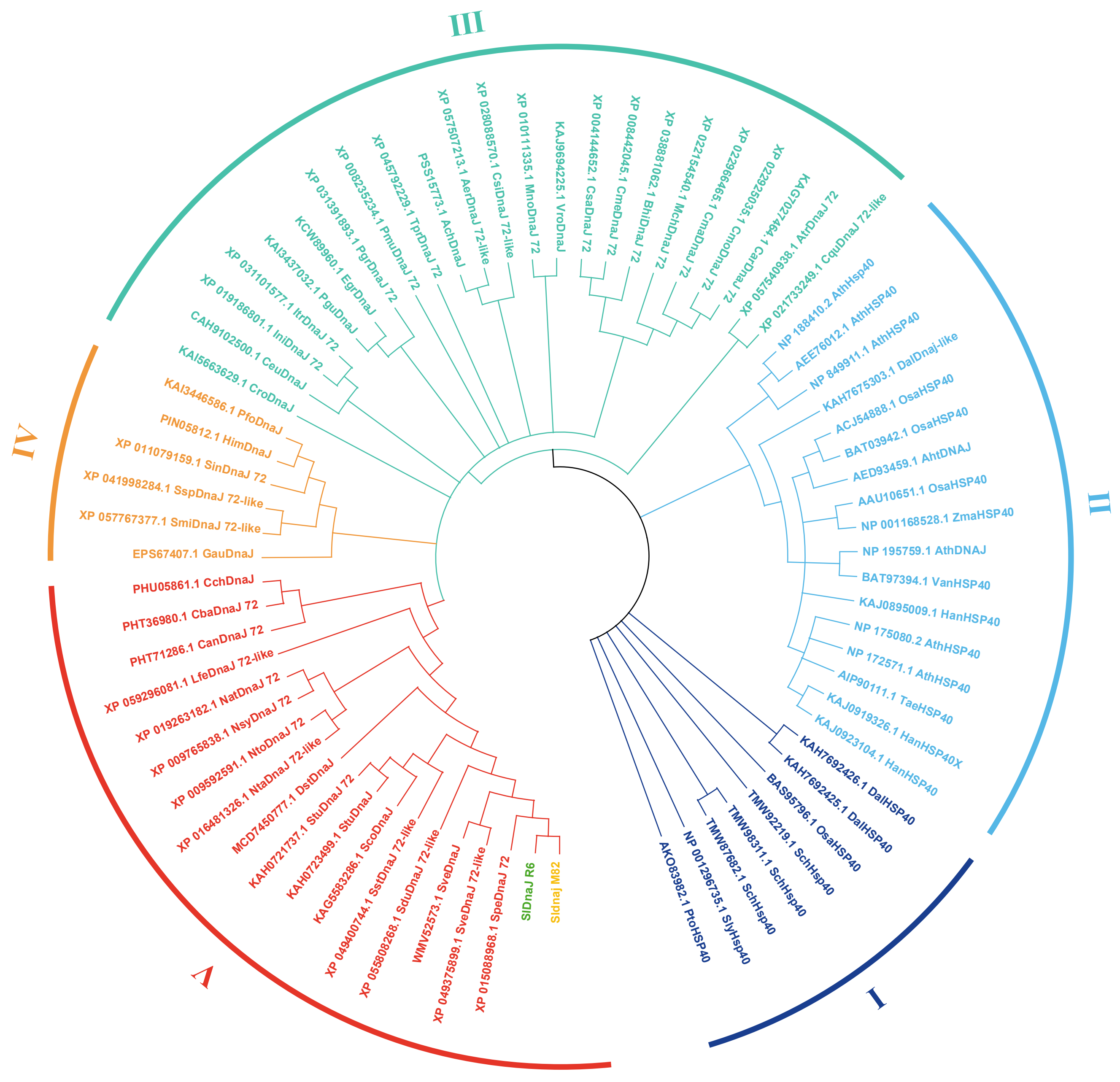

Supplement: Web_Material_uhaf019 [file web_material_uhaf019.zip › Fig. S8 revised.pdf]

A

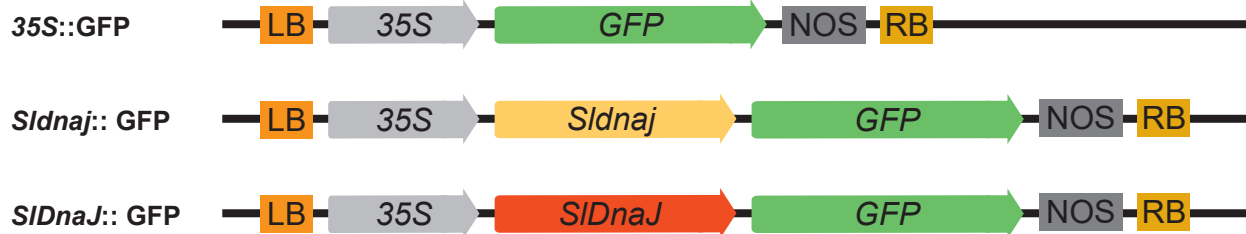

B

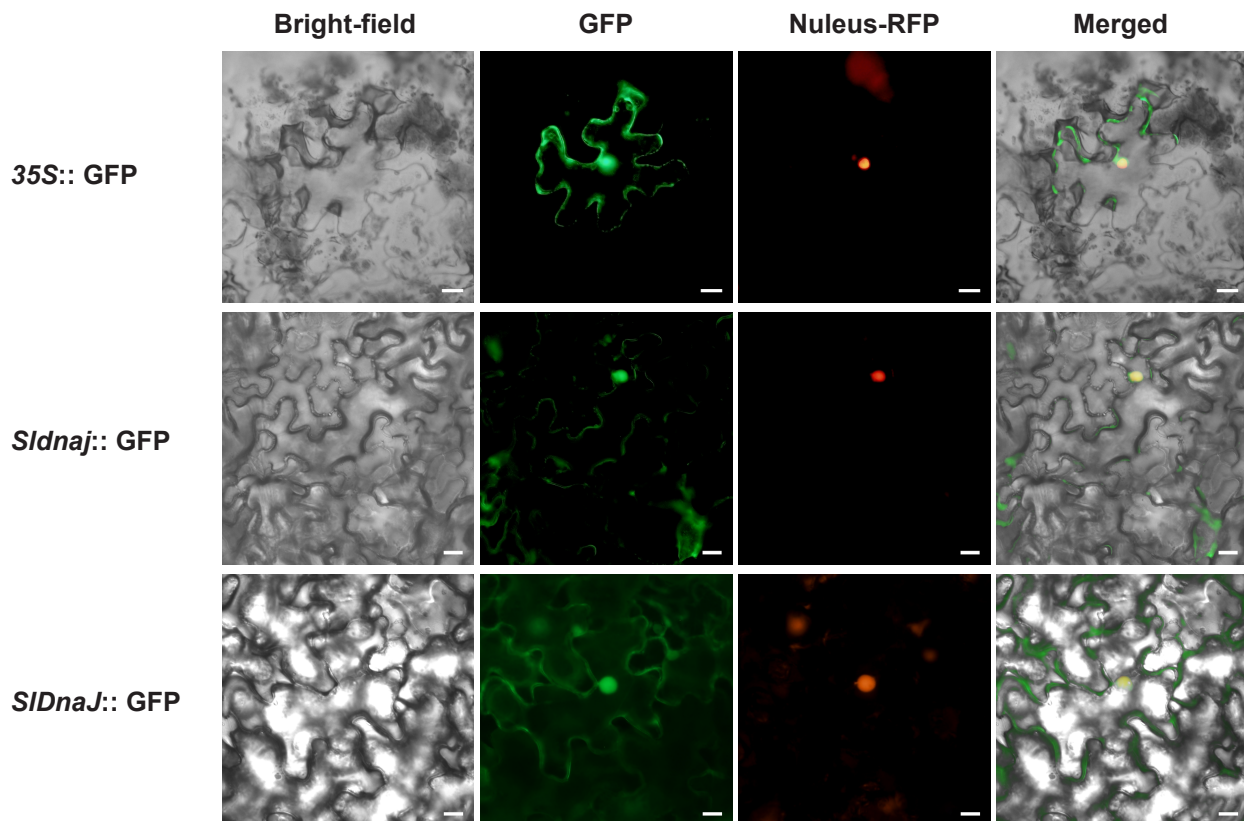

Supplement: Web_Material_uhaf019 [file web_material_uhaf019.zip › Fig. S9.pdf]

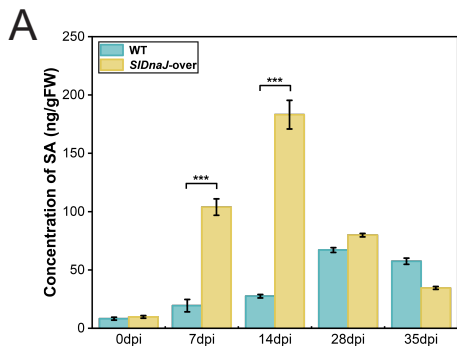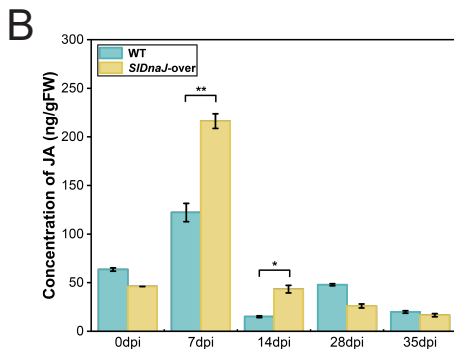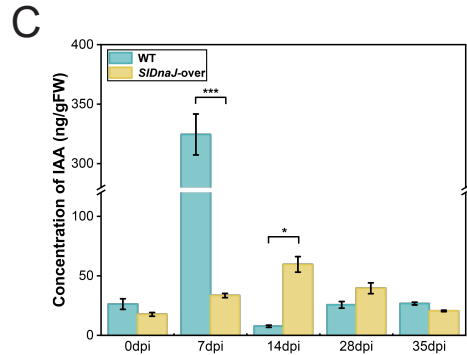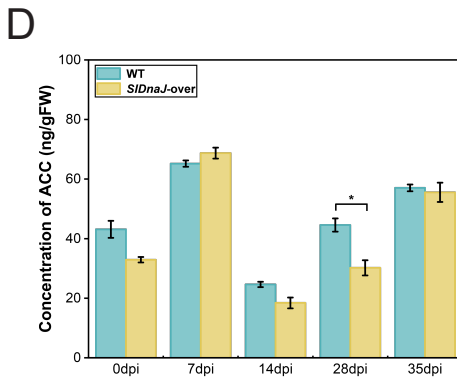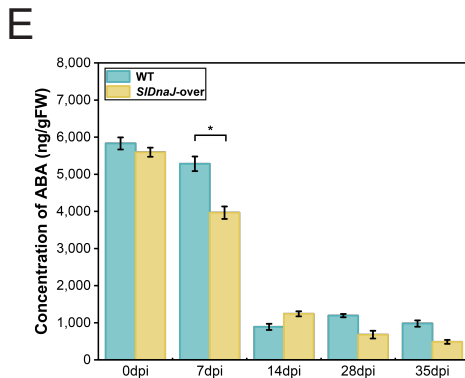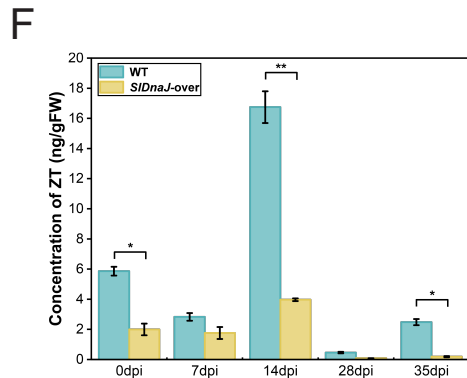

Supplement: Web_Material_uhaf019 [file web_material_uhaf019.zip › Fig. S10.pdf]

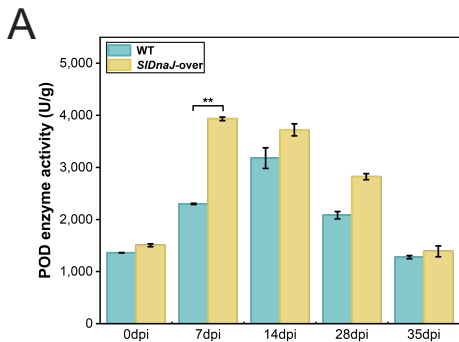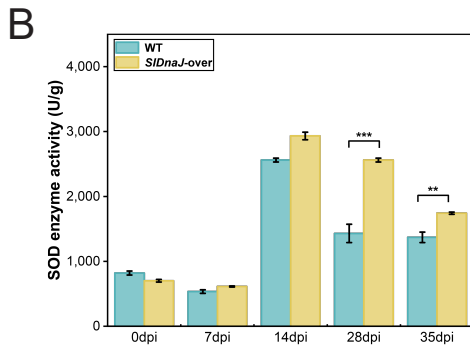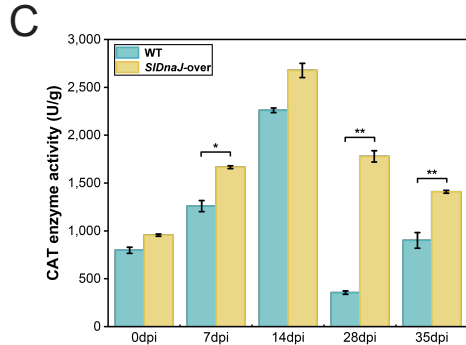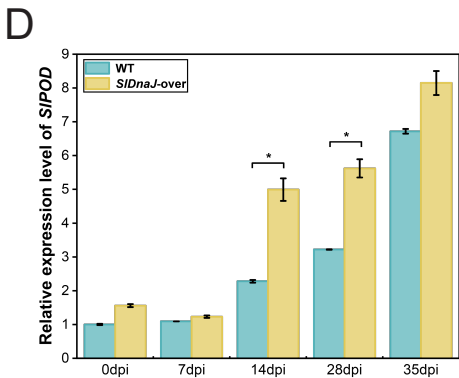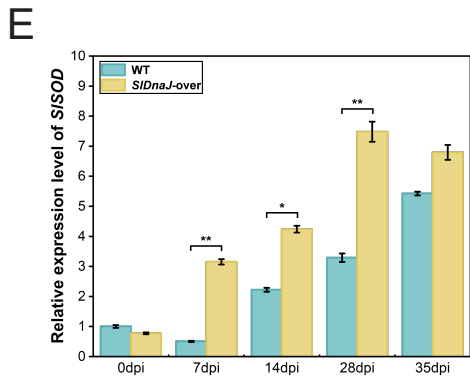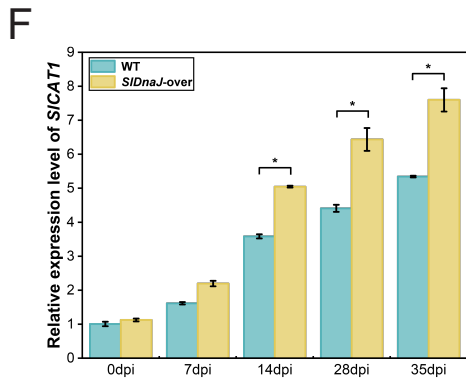

Supplement: Web_Material_uhaf019 [file web_material_uhaf019.zip › Fig. S11.pdf]

**A**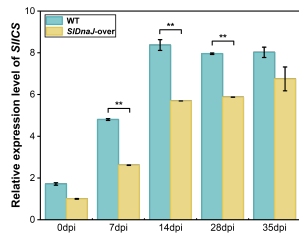**B**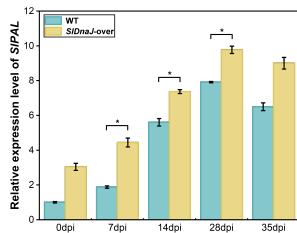**C**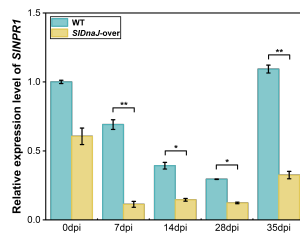**D**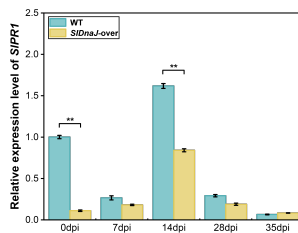**E**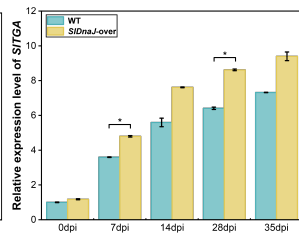**F**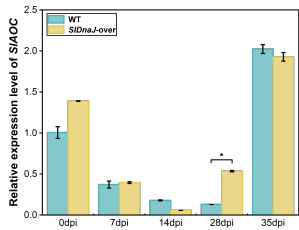**G**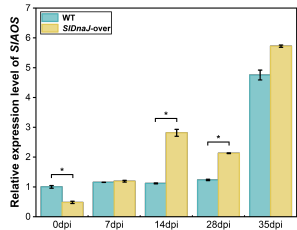**H**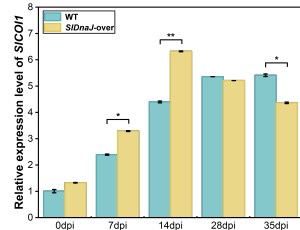**I**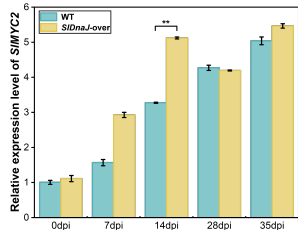**J**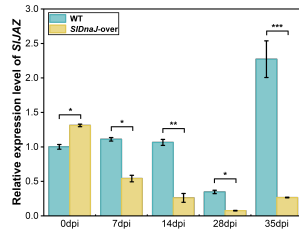

Supplement: Web_Material_uhaf019 [file web_material_uhaf019.zip › Fig. S12.pdf]

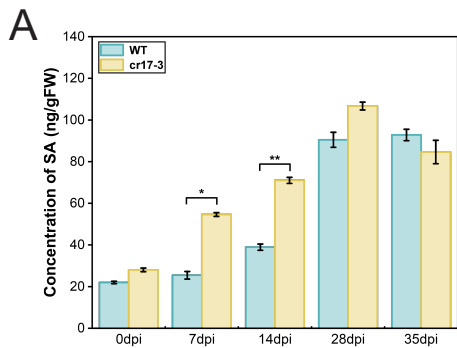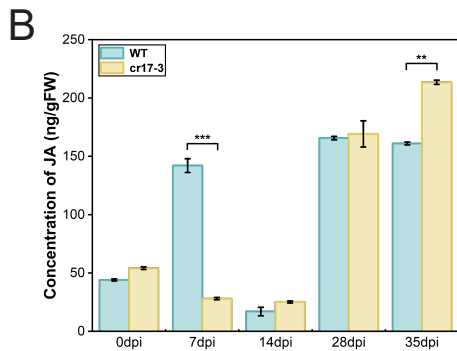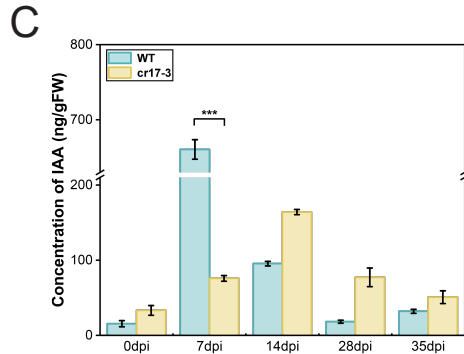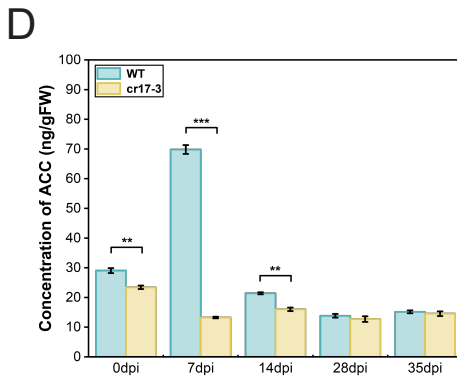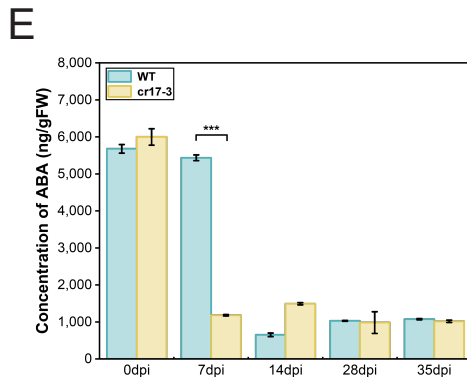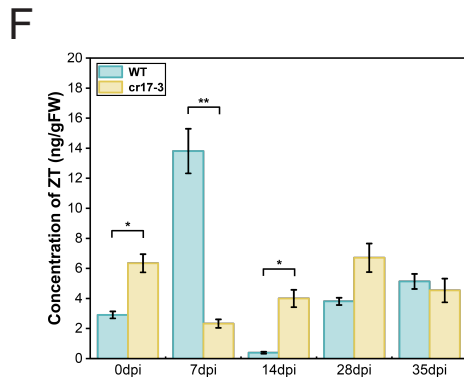

Supplement: Web_Material_uhaf019 [file web_material_uhaf019.zip › Fig. S13.pdf]

**A**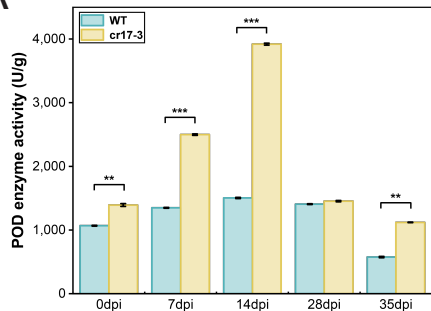**B**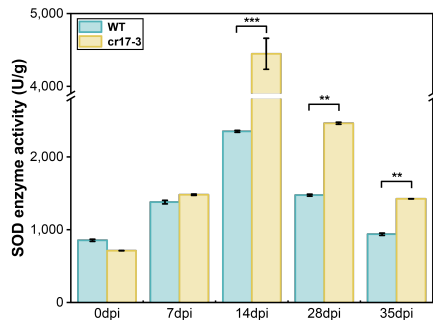**C**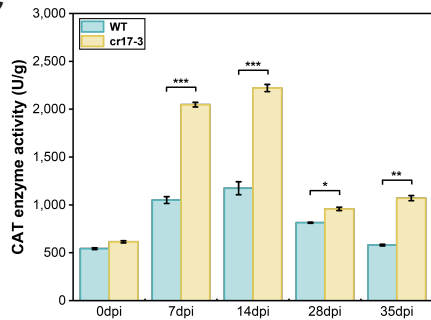**D**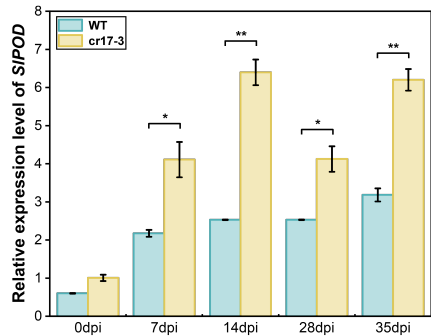**E**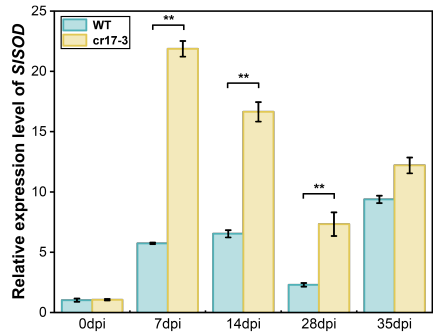**F**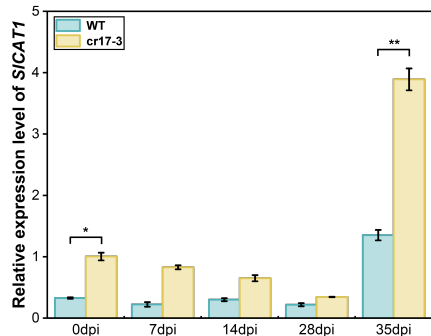

Supplement: Web_Material_uhaf019 [file web_material_uhaf019.zip › Fig. S14.pdf]

**A**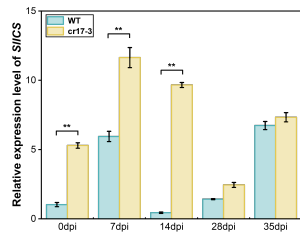**B**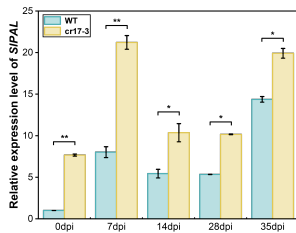**C**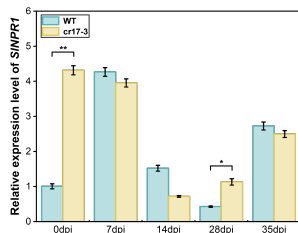**D**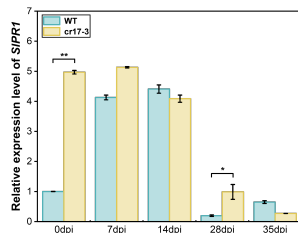**E**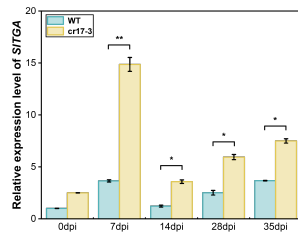**F**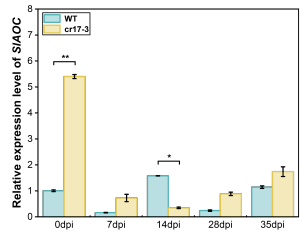**G**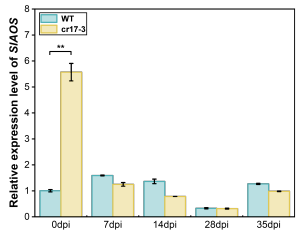**H**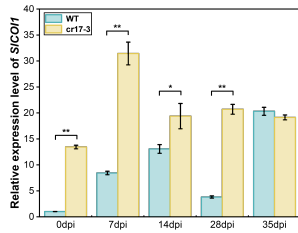**I**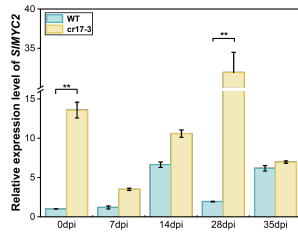**J**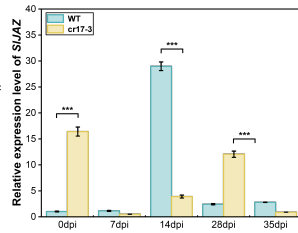

Supplement: Web_Material_uhaf019 [file web_material_uhaf019.zip › Fig. S15.pdf]
